# Non‐pituitary growth hormone enables colon cell senescence evasion

**DOI:** 10.1111/acel.14193

**Published:** 2024-05-09

**Authors:** Vera Chesnokova, Svetlana Zonis, Tugce Apaydin, Robert Barrett, Shlomo Melmed

**Affiliations:** ^1^ Department of Medicine Cedars‐Sinai Medical Center Los Angeles California USA; ^2^ Board of Governors Regenerative Medicine Institute Cedars‐Sinai Medical Center Los Angeles California USA

**Keywords:** DNA damage, growth hormone, senescence‐associated secretory phenotype

## Abstract

DNA damage‐induced senescence is initially sustained by p53. Senescent cells produce a senescence‐associated secretory phenotype (SASP) that impacts the aging microenvironment, often promoting cell transformation. Employing normal non‐tumorous human colon cells (hNCC) derived from surgical biopsies and three‐dimensional human intestinal organoids, we show that local non‐pituitary growth hormone (npGH) induced in senescent cells is a SASP component acting to suppress p53. npGH autocrine/paracrine suppression of p53 results in senescence evasion and cell‐cycle reentry, as evidenced by increased Ki67 and BrdU incorporation. Post‐senescent cells exhibit activated epithelial‐to‐mesenchymal transition (EMT), and increased cell motility. Nu/J mice harboring GH‐secreting HCT116 xenografts with resultant high GH levels and injected intrasplenic with post‐senescent hNCC developed fourfold more metastases than did mice harboring control xenografts, suggesting that paracrine npGH enables post‐senescent cell transformation. By contrast, senescent cells with suppressed npGH exhibit downregulated Ki67 and decreased soft agar colony formation. Mechanisms underlying these observations include npGH induction by the SASP chemokine CXCL1, which attracts immune effectors to eliminate senescent cells; GH, in turn, suppresses CXCL1, likely by inhibiting phospho‐NFκB, resulting in SASP cytokine downregulation. Consistent with these findings, GH‐receptor knockout mice exhibited increased colon phospho‐NFκB and CXCL1, while GH excess decreased colon CXCL1. The results elucidate mechanisms for local hormonal regulation of microenvironmental changes in DNA‐damaged non‐tumorous epithelial cells and portray a heretofore unappreciated GH action favoring age‐associated epithelial cell transformation.

## Graphical abstract

Growth hormone (GH), stimulated by DNA damage and CXCL1, is induced in senescent cells and is a component of senescence‐associated secretory phenotype (SASP). SASP GH triggers senescence evasion in a subset of senescence cells and activates epithelial‐to‐mesenchymal transition (EMT), resulting in post‐senescent cell migration and transformation.

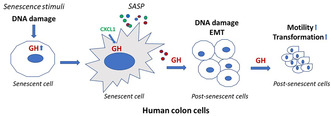

**Abbreviations**
ATM: ataxia telangiectasia mutated
ATR: ataxia telangiectasia and Rad3‐related
BRCA: breast cancer protein
BrdU: bromodeoxyuridine
Cdk: cyclin‐dependent kinase
CS: Cockayne syndrome
CSB: Cockayne syndrome group B
CXCL1: chemokine (C‐X‐C motif) ligand 1
CXCL2: C‐X‐C motif chemokine ligand 2
CXCL5: C‐X‐C motif chemokine ligand 5
CXCR2: C‐X‐C chemokine receptor 2
DMSO: dimethyl sulfoxide
ELISA: enzyme‐linked immunosorbent assay
EMT: epithelial‐to mesenchymal transition
GFP: green fluorescent protein
GH: growth hormone
GHR: growth hormone receptor
GHRKO: GH receptor knockout
HGPS: Hutchinson–Gilford progeroid syndrome
hNCC: human normal colon cells
IGF1: insulin‐like growth factor 1
IL1: interleukin 1
iPSC: induced pluripotent stem cells
JAK2: Janus kinase 2
Lenti‐shGH RNA: lentivirus expressing short hairpin GH RNA
Lenti‐shScr: lentivirus expressing short hairpin scramble (control) RNA
LGR5: leucine rich repeat containing G protein coupled receptor 5
NFκB: nuclear factor kappa‐light‐chain‐enhancer of activated B cells
npGH: non‐pituitary growth hormone
PCNA: proliferating cell nuclear antigen
Rel‐A: Rel protooncogene A, NFκB subunit
SASP: senescence associated secretory phenotype
SA‐β‐gal: senescence‐associated β galactosidase
SOX2: sex determining region Y‐box 2
STAT: signal transducer and activator of transcription
UV: ultraviolet light
UVC: ultraviolet C light
HRAS: Harvey rat sarcoma virus oncogene
XP: xeroderma pigmentosa

## 1 INTRODUCTION

Pituitary growth hormone (GH) and its mediator insulin‐like growth factor 1 (IGF1) induce skeletal growth and also elicit anabolic and metabolic functions in adults (Devesa et al., 2016; Kopchick et al., 2020; Rosenfeld & Hwa, 2009; Waters & Brooks, 2012). GH signals through the GH receptor (GHR) dimer via JAK2 to initiate pleiotropic distal cell‐specific responses (Rosenfeld & Hwa, 2017; Waters, 2016). However, mechanisms underlying mature adult GH functions are largely enigmatic, and signaling pathways mediating adult GH actions on cell proliferation are not well delineated.

While circulating GH declines with age (Bartke, 2017, 2020; Bartke et al., 2016; Colon et al., 2019; Masternak & Bartke, 2012), local non‐pituitary GH (npGH) is induced with aging in colon tissue (Chesnokova et al., 2021) and in senescent cells (Chesnokova et al., 2013). Aging, associated with telomere shortening and increased DNA damage, leads to senescence, which also occurs in response to genotoxic stressors, followed by depletion of viable functioning cells (McHugh & Gil, 2018; Sharpless & Sherr, 2015). Unrepaired DNA damage induced by radiation, chemotherapy, and oxidative stress, as well as some activated oncogenes, triggers senescence (Di Micco et al., 2021) thereby restraining uncontrolled proliferation of potentially tumorigenic cells (Gorgoulis et al., 2019; Sharpless & Sherr, 2015).

Aged human (Chkhotua et al., 2003; Dimri et al., 1995; Liu et al., 2009) and murine (Burd et al., 2013; Wang et al., 2009; Yousefzadeh et al., 2020) tissues accumulate senescent cells. With age, DNA damage response and repair pathways are attenuated (Goukassian et al., 2000; Gutierrez‐Martinez et al., 2018); in turn, increased numbers of senescent cells with unrepaired DNA damage contribute to age‐associated decline of tissue function by adversely impacting the cell microenvironment (Childs et al., 2017), ultimately leading to decreased lifespan. Although senescence has been defined as a state of irreversible proliferation arrest, more evidence has emerged that senescence can be reversed in both non‐tumorous (Ei et al., 2021; Martin et al., 2014; Romanov et al., 2001) and tumorous cells (De Blander et al., 2021; Gosselin et al., 2009; Saleh et al., 2019). As DNA damage is usually unrepaired in senescent cells (Childs et al., 2017), these cells may be prone to acquiring oncogenic mutations and transformation if they proliferate. Mechanisms underlying senescence evasion are not well understood.

Senescent cells, characterized by high expression of Cdk inhibitors p16INK4a, p21(Cip1), and p27(Kip1), exit the cell cycle and acquire a senescence‐associated secretory phenotype (SASP). These cells secrete matrix metalloproteinases that degrade extracellular matrix, as well as cytokines and chemokines, including chemokine C‐X‐C motif ligand 1 (CXCL1), interleukin (IL)‐1β, IL6, IL8, and vascular endothelial growth factor A, all of which may act in autocrine and paracrine manners. Thus, senolytics were recently proposed to treat age‐related disorders (Chaib et al., 2022; Kirkland & Tchkonia, 2020).

SASP composition depends on pro‐senescence inducers (Basisty et al., 2020; Childs et al., 2015) as well as on the SASP‐producing cell type (Lee & Schmitt, 2019). SASP factors are pleiotropic, and may reduce or induce proliferation in neighboring cells and alter tissue homeostasis (Sharpless & Sherr, 2015). SASP attracts immune effectors, leading to senescent cell depletion (Kang et al., 2011; Lee & Schmitt, 2019; Xue et al., 2007), and secondary senescence induction in neighboring cells (Acosta et al., 2013; Kuilman & Peeper, 2009). Furthermore, SASP either induces (Demaria et al., 2014; Krtolica et al., 2001) or constrains (Acosta et al., 2013; Xue et al., 2007) tumor cell proliferation, and may also promote preneoplastic cell transformation that often occurs in aging tissues (Calcinotto et al., 2019; Lee & Schmitt, 2019). Thus, suppressing SASP by eliminating senescent cells or blocking the secretome may ameliorate adverse age‐related microenvironment changes (Baker et al., 2011; Childs et al., 2015).

While known SASP factors are not uniquely specific to senescent cells, most senescent cells express p16 and are characterized by increased senescence‐associated β‐galactosidase (SA‐β‐gal) activity. Little is known about molecular mechanisms modulating SASP content and paracrine SASP effects, and few studies have elucidated SASP actions in a non‐tumorous environment.

npGH, synthesized locally in peripheral tissues (Chesnokova et al., 2013; Chesnokova, Zonis, Barrett, Kameda, et al., 2019; Devesa et al., 2016; Perry et al., 2017; Render et al., 1995), is identical to endocrine GH1 produced by the pituitary and acts through autocrine/paracrine mechanisms through the widely expressed GHR (Rosenfeld & Hwa, 2017) that recognizes both pituitary GH and npGH ligands. As we have shown that npGH is markedly upregulated in DNA damage‐induced senescent cells (Chesnokova et al., 2013, 2021; Chesnokova, Zonis, Barrett, Kameda, et al., 2019), we investigated whether npGH is universally induced in different senescence types. Employing normal human non‐tumorous colon cells (hNCC) derived from surgical biopsies, we show here that npGH is induced in response to oncogene‐, therapy‐, UV radiation‐, and/or replicative‐induced senescence and likely constitutes a SASP component. Induced paracrine‐acting npGH, consistent with a SASP function, signals to neighboring cells, triggering proliferation and senescence evasion, suppressing DNA damage repair proteins, and increasing DNA damage.

Moreover, we show that npGH suppresses E‐cadherin, associated with cell‐to‐cell adhesion, and activates Twist2, an epithelial‐to‐mesenchymal transition (EMT) marker in post‐senescent cells. These observations were affirmed in vivo when immunocompromised mice harboring GH‐secreting HCT116 xenografts and injected intrasplenic with post‐senescent hNCC developed significantly more metastases than did mice harboring control vector‐expressing xenografts.

In determining a mechanism for our findings, we also show that CXCL1, the human ortholog of IL8 and a key SASP component, induces npGH. We found that CXCL1, highly abundant in models of colon senescence, induces npGH expression. In turn, npGH suppresses CXCL1 and other SASP components, thus modifying SASP and reversing the senescence phenotype, likely by suppressing activation of NFκB, a transcription factor for CXCL1 and cytokines (Burke et al., 2014; Liu et al., 2017). Consistent with these findings, we also show that colon CXCL1 is suppressed in vivo in mice with excess circulating GH, while both CXCL1 and phospho‐NFκB are induced in *GHR* knockout (GHRKO) mice with abrogated GH signaling.

Our results suggest that epithelial npGH induced by CXCL1 constitutes a SASP component that suppresses p53, enabling senescent cell proliferation and transformation, and acts in a paracrine manner to promote DNA damage in neighboring cells. Our study thus sheds light on mechanisms underlying hormone‐mediated microenvironmental changes in non‐tumorous epithelial cells after DNA damaging therapy or with age.

## 2 MATERIALS AND METHODS

### 2.1 Mice

Athymic nude female Nu/J and GHRKO mice were purchased from The Jackson Laboratory. As breeding was undertaken with heterozygous males and females, WT and GHRKO mice were obtained from the same breeding. Heterozygous mice were backcrossed with WT mice at least six times. Twenty‐four‐month‐old females and males were used for experiments as indicated.

### 2.2 Rats

To study effects of local colon GH signaling suppression, 10 hypophysectomized male rats (HYPOX, Charles River) were used. Each rat weighted approximately 200 g. All rats were injected daily for 5 days. There were three groups in the study: (1) three control rats were injected intraperitoneally with 200 μL of 5% dextrose and subcutaneously with 80 μL of PBS; (2) four rats were injected intraperitoneally with 4 mg/kg oxaliplatin (Sigma, cat# O9512) in 5% 200 μL dextrose, and subcutaneously with 80 μL of PBS as control for pegvisomant; (3) three rats were injected with 4 mg/kg oxaliplatin in 5% 200 μL dextrose and subcutaneously with 0.57 mg/kg of pegvisomant (Somavert, Pfizer) in 80 μL of PBS. Animals were sacrificed 3 days after the last injection.

### 2.3 3D human intestinal organoids

Generation and culturing of intestinal organoids from fibroblast‐derived induced pluripotent stem cells (IPSCs) were as described (Barrett et al., 2014; Workman et al., 2018). Three‐dimensional intestinal organoids were generated from iPSC lines using episomal plasmid reprogramming. To induce definitive endoderm formation, iPSCs were cultured with activin A (100 ng/mL; R&D Systems) with increasing concentrations of FBS (0%, 0.2%, and 2% [vol/vol] on Days 1, 2, and 3, respectively). Wnt3A (25 ng/mL; R&D Systems) was also added on the first day of endoderm differentiation. To induce hindgut formation, cells were cultured in Advanced DMEM/F12 with 2% (vol/vol) FBS along with CHIR 99021 (2 μM; Tocris) and FGF4 (500 ng/mL; R&D Systems). After 3–4 days, free‐floating epithelial spheres and loosely attached epithelial tubes were harvested. Epithelial structures were subsequently suspended in Matrigel (Corning, cat#354248) and overlaid in intestinal medium containing CHIR99021 (2 μM; Tocris), noggin and EGF (both 100 ng/mL; R&D Systems), and B27 (1×; Invitrogen). Organoids were sorted for EPCAM, an epithelial cell marker, 30 days after differentiation and passaged every 7–10 days thereafter. Intestinal organoids were validated after identifying enterocytes, goblet cells, Paneth cells, and enteroendocrine cells as well as expression of CDX2 (Gao et al., 2009).

### 2.4 Cells and treatments

Human normal colon cell (hNCC) line #1 and line #2 (Applied Biological Materials, lot#HC1211 and lot#0145834955002, respectively) were obtained from de‐identified normal colon sections of two individuals and validated as cytokeratin 18‐ and 19‐expressing colon epithelial cells. Donor sex and age were not available. Cells were cultured in PriGrow III Medium (Applied Biological Materials) supplemented with 5% FBS, and with antibiotic/antimycotic solution (Gemini Bio‐Products), then infected or treated before Passage 5. Except as specifically mentioned, all experiments were conducted in hNCC line #1.

MCF12A and HCT116 were purchased from ATCC. MCF12A were cultured in DMEM/F12 medium (Invitrogen) with 0.5 μg/mL hydrocortisone (MilliporeSigma), 10 μg/mL insulin (MilliporeSigma), 20 ng/mL EGF (Invitrogen), and 5% horse serum (Omega Scientific). HCT116 were cultured in McCoy 5A medium (Invitrogen) and 10% FBS.

Normal human primary epidermal keratinocytes (ATCC, cat#PCS‐200‐011) were grown in Dermal Basal Medium with Keratinocyte Growth Kit (ATCC, cat#PCS‐200‐030 and #PCS‐200‐040, respectively).

Normal human colon fibroblasts (Cell Biologics) were cultured in Complete Fibroblast Medium Kit with Supplement (Cell Biologics, cat#M2267). Cells from Passage 3 were used for experiments.

Human skin fibroblasts derived from patients with progeroid syndrome or healthy volunteers were purchase from Coriell Institute NIGMS human genetic cell repository and cultured in Complete Fibroblast Medium (Cell Biologics).

Etoposide (MilliporeSigma, cat#E1383) was prepared as a 50 mM DMSO stock solution. Nutlin3 (MilliporeSigma, cat#N6287) was prepared in 20 mM DMSO stock solution and cells treated with 3 μM for 72 h. Control cells were treated with appropriate dilutions of DMSO. Human recombinant CXCL1 was purchased from BioLegend (cat#574404). Human recombinant GH was purchased from BioVision (cat#4769), and used as 500 ng/mL in 0.1% BSA in PBS, a conventional dose for in vitro studies (Basu et al., 2017; Qian et al., 2020; Schneider et al., 2019).

UVC cell irradiation was performed with Phillips germicidal UV lamp that delivers UVC radiation at 254 nM wavelength. Radiation intensity was measured with Extech Instruments UVA, UVC light meter (model SDL470). Cells were plated into a 10 cm dish and, after reaching 80%–90% confluency, medium was rinsed, and 6 mL PBS added. Cells were irradiated in open dishes for 2 s to reach a radiation dose of 1 J/m^2^ and for 4 s to reach 2 J/m^2^. After irradiation, PBS was substituted with full medium and cells incubated for the indicated times.

To establish post‐senescent cells, hNCC were treated with 50 μM of etoposide for 48 h, then stained with Cellular Senescence Live Cell Analysis Assay Kit from Enzo (cat#ENZ‐Kit 130‐0010) overnight, and senescent cells sorted for SA‐β‐gal with AriaIII cell sorter and plated. Cells were cultured for an additional 10 days in the presence or absence of 500 ng/mL GH, and cells were sorted again for SA‐β‐gal. Approximately 85% cells became SA‐β‐gal‐negative, defining them as post‐senescent cells for analysis.

### 2.5 Mouse xenografts

HCT116 cells stably infected with lentivirus‐expressing murine GH (lenti‐mGH) or empty vector (lentiV) (5 × 10^5^ cells in 0.05 mL PBS) were mixed (1:1) with High Concentration Matrigel Matrix (Corning, cat#354263) and injected into the right flank of nude athymic female mice to establish a model of excess systemic GH. Mice were sacrificed 5 weeks after injection. Circulating levels of GH in lenti‐mGH mice were measured by murine GH ELISA (Millipore) as described (Chesnokova et al., 2016).

To establish the ability of post‐senescent hNCC cells to metastasize in the presence of human GH, 5‐week‐old nude female mice were injected subcutaneously with 5 × 10^5^ HCT116 cells stably transfected with lentivirus‐expressing human GH (lentiGH) or vector (lentiV) in PBS/Matrigel. Mice developed subcutaneous tumors after 3 weeks, and then 2 × 10^6^ post‐senescent SA‐β‐gal‐negative hNCC in 100 μL of PBS were injected intrasplenic to induce metastasis. Animals were sacrificed 3 weeks later and number and size of metastatic lesions were assessed. Ten mice/group were used, but two mice in each group failed to develop subcutaneous xenografts after 3 weeks and were excluded from the study.

Levels of circulating human GH in mice with xenografts were measured with hGH ELISA kit (ALPCO, cat#25‐HGHHU‐E01), and murine IGF1 was measured with mouse/rat IGF1 ELISA kit (ALPCO, cat#22‐IG1MS‐E01).

### 2.6 Constructs and transfections

Lentiviral particles expressing hGH (pLV‐EF1p‐hGH1‐IRES‐eGFP‐WPRE) and respective control lentiviral particles (pLV‐EF1p‐mCherry‐IRES‐eGFP‐WPRE), as well as murine GH (EF1‐luc2‐GH‐Ubic) and vector (EF1‐luc2‐Ubic) were generated at the Regenerative Medicine Institute at Cedars‐Sinai. V‐12HRAS (cat#G221, stock 3.69 × 10^7^ IU/mL) and lenti‐CMV control vector (cat#LVP690, stock 1.42 × 10^8^ IU/mL) were purchased from Applied Biological Materials. Lentiviral particles expressing human GH shRNA, CXCL1 shRNA, and control shRNA Scr lentiviral particles were all from Santa Cruz Biotechnology (cat#sc‐43803‐V, #sc‐43816‐V, and #sc‐108080, respectively) and were received as stock solutions (1 × 10^6^ IU/200 μM in DMEM).

hNCC or HCT116 were plated 1 day before transfections and were infected with 50 MOI lentivirus particles and 8 μg/mL polybrene added.

Organoids were dispersed into single‐cell suspension using TrypLE (Gibco, cat#12604) and mixed with Matrigel together with 50 MOI lentiviral particles and 8 μg/mL polybrene. Cells in the Matrigel bubble were plated into 24‐well plates and overlaid with intestinal organoid media enriched with 10 nM Rock inhibitor (Tocris, cat#1254), 500 nM A8301 (Tocris, cat#2939), and 10 μM SB202190 (Tocris, cat#1254).

### 2.7 Cocultures

For coculture experiments using hNCC, 100,000 hNCC per well were plated into 6‐well plates with 24 mm inserts and 0.4 μm pore size (Corning, cat#3412), and 300,000 lentiGH or lentiV hNCC were plated into inserts in 1 mL of media. Cells were cocultured for 4 days, after which inserts with lentiGH or lentiV hNCC were removed and cells growing on the bottom of 6‐well plates were either scratched with a cell scraper and removed for comet assay or pulsed with BrdU (Roche) for 1 h, collected by trypsinization, fixed in glycine‐ethanol fixative, and stained for BrdU with BrdU labeling and detection kit (Roche, cat#11296736001). A minimum of 50,000 cells were analyzed by FACS using FlowJo software.

For coculture experiments assessing BrdU incorporation and DNA damage using intestinal organoids, organoid‐derived cells were transduced with lentiGH or lentiV. One week after transduction, lentiGH or lentiV organoids were plated in 12‐well plates in a Matrigel bubble on the left part of each well, and non‐treated organoids plated in a Matrigel bubble on the right part of the same well. Matrigel bubbles were overlaid with o.5 mL intestinal organoid media and organoids were cocultured for 4 days. The right bubble containing non‐transduced cells was then scratched and cells removed for Comet assay, or BrdU was added into media for 2 h and Matrigel bubbles collected for BrdU proliferation assay.

### 2.8 Protein analysis

For western blot analysis, cells were homogenized and lysed in RIPA buffer (Cell Signaling, cat#9806S) with protease inhibitors (MilliporeSigma, cat#P8340) or isolated from TRIzol (Ambion, cat#15596018) and dissolved in 1% SDS according to the protocol for RNA/DNA/protein isolation from Molecular Research Center, Inc. Proteins were separated by SDS‐PAGE, electroblotted onto Trans‐Blot Turbo Transfer Pack 0.2 μm PVDF membrane (BioRad), and incubated overnight with antibodies, followed by corresponding secondary antibodies (Amersham). To detect low abundance proteins, Hikari signal enhancer kit was used (Nacalai USA; cat#NU00102).

For detection of GH and CXCL1 in the media, cells were treated with etoposide and 6 days later 1 × 10^6^ senescent cells were collected and re‐plated from each group. Media used for western blotting was collected 24 h after replating. Media was spun at 1000 rpm for 10 min to remove floating cells and debris, and filtered through 0.2 μm Tuffryn membrane syringe filter (Life Sciences, cat#4612). Protease inhibitors were then added, and media mixed 1:1 with BioRad Laemmli Buffer (cat#1610737).

The following primary antibodies were used: GAPDH and p16 from Santa Cruz Biotechnology (cat #sc‐25778 and #sc‐1661, respectively); β‐galactosidase and CXCR2 from Lifespan Biosciences (cat#LS‐B10989 and #LS‐C388292, respectively); and GH from R&D Systems (cat#AF1067) or Lifespan Biosciences (cat#LS‐B4199). β actin was from MilliporeSigma (cat#A1978), p53 from R&D Systems (cat#AF1355), CXCL1 and Ki67 from Abcam (cat#ab86436 and #ab15580, respectively), and V12‐HRAS antibody from Invitrogen (cat#RAS‐78086). phospho‐STAT (Tyr694, cat#4322), phospho‐NFκBp65 (Ser536, cat#3033), and total NFκBp65 (cat#8242) were from Cell Signaling. Phospho‐BRCA1 (Ser1524), phospho‐ATR (Ser428), and phospho‐ATM (Ser1981) were from Cell Signaling (cat#9009, #2853, and #13050, respectively), phospho‐BRCA2 (Ser2095) from Invitrogen (cat#PA5‐105585), and Twist2 from Santa Cruz Biotechnology (cat#sc‐81417). IL1β and IL6 were from Abcam (cat#ab2105 and #ab6672, respectively).

GH was detected in culture medium using human GH ELISA kit from Abcam (cat#ab190811).

### 2.9 Immunocytochemistry

In some experiments, after performing SA‐β‐gal enzymatic assays, cells in 6‐well plates were washed in PBS and stained with Ki67 (Abcam, cat#ab15580, dilution 1:1000) or GH (Lifespan Biosciences, cat#LS‐C4199, dilution 1:200) antibody using ImmPress Excell Staining Kit Peroxidase from Vector (cat#MP‐7601). Senescent and double‐positive SA‐β‐gal and Ki67 cells were counted at ×200 magnification in 20–30 fields/well.

### 2.10 Comet assay

The level of DNA damage in each individual cell was measured by analyzing accumulation of DNA breaks with OxiSelect Comet Assay kit (cat#STA350) per manufacturer instructions. Single‐cell alkaline electrophoresis was used for 30 min at 1 volt/cm. DNA damage (intensity of the staining) was quantified by ImageJ according to the manual as percent of damaged DNA in the tail of the entire cell DNA multiplied by the length of the tail (Olive tail moment = tail DNA% × tail moment length). Data collected from at least three independent experiments and at least 200 nuclei/group were analyzed.

### 2.11 Real‐time PCR

Total RNA was isolated with TRIzol (Ambion, cat#15596018) followed by RNAeasy mini Kit (Qiagen). After DNAse I treatment (TURBO DNA free, Ambion), cDNA was synthesized from 1 μg purified RNA by the SuperScript II First‐Strand cDNA synthesis system (Thermo Fisher). Quantitative PCR was performed in 20 μL reactions using IQ SYBR Green Master Mix in BioRad IQ5 instrument (BioRad Laboratories). Specific validated primers for human GH1 were as described (Chesnokova et al., 2016). IGF1, CXCL1, and GAPDH were purchased from SuperArray (Qiagen). Experiments were performed in triplicate. Relative mRNA quantities in experimental samples were determined by CFX Maestro 1.0 software expressed in arbitrary units as fold‐difference from control.

### 2.12 Telomere length

Telomere length was measured by Relative Telomere Length Quantification qPCR Assay. DNA was isolated from TRIzol (Molecular Research Center, Inc.), and 5 ng genomic DNA per reaction used. Reactions were conducted in triplicate. Relative telomere length in experimental samples was determined by CFX Maestro 1.0 software, normalized to the expression of single copy reference 100‐bp long region of human chromosome 17 and expressed in arbitrary units as fold‐difference from control.

### 2.13 SA‐β‐galactosidase assay

SA‐β‐gal enzymatic activity was assessed in vitro using Senescence Cell Histochemical Staining Kit (Sigma‐Aldrich, cat#CS0030) as described (Chesnokova et al., 2021). Briefly, cells were plated in 6‐well plates, treated for the indicated times, washed with PBS (pH 6.0), fixed, and stained with 5‐bromo‐4‐chloro‐3‐indolyl‐h‐D‐galactopyranoside (X‐Gal) overnight at 37°C. Only senescent cells stain at pH 6.0.

Organoids were dissociated into single cell suspensions incubated in TrypLE Express (Gibco, cat#12604‐021) and plated in intestinal organoid media into 12‐well plates pretreated with Enactin‐collagen IV‐laminin (ECL) Cell Attachment Matrix (Millipore, cat#08‐110). After 48 h, enzymatic reactions were performed with Senescence Cell Histochemical Staining Kit as above.

### 2.14 Sorting for SA‐β‐galactosidase

To select senescent cells, cells were stained overnight using Cellular Senescence Live Cell Analysis Assay Kit from Enzo (cat#ENZ‐KIT 130‐0010) and sorted at excitation/emission: 480/520 nm using Aria III cell sorter. After sorting, cells were cultured and for some experiments, pulsed with BrdU, as described below.

### 2.15 Soft agarose assay

shScr or shGH hNCC were plated and treated with 50 μM etoposide in complete media. After 48 h, cells were washed and maintained without drug for an additional 3 days, then stained overnight for SA‐β‐gal expression with Cellular Senescence Live Cell Analysis Assay Kit according to the manual. Staining was performed overnight, and cells sorted for green fluorescence on the Aria III sorter.

In total, 20,000 senescent cells/well were plated into 6‐well plates in duplicate in full PriGrow media in 0.3% agarose on top of the lower agarose layer (0.6%) with or without 500 ng/mL GH. After 48 h, 1 mL of fresh media with or without GH was carefully layered over 0.3% agarose; media was changed every other day. After 16 days, colonies were stained with MTT for 1 h and counted. Experiment was conducted in duplicate.

### 2.16 Cell proliferation assay

Asynchronized cells were pulsed with BrdU using BrdU Labeling and Detection Kit from Roche (cat#11299964001), diluted 1:1000 in medium with 5% FBS, and cultured for 1 h at 37°C. Cells were washed in PBS, trypsinized, fixed in glycine/70% ethanol fixative at pH 2.0 for 1 h at 20°C, and immunostained with anti‐BrdU mouse monoclonal antibody (BrdU Labeling and Detection Kit) for 30 min at 37°C, followed by donkey anti‐mouse Alexa Fluor 488 secondary antibody for 1 h at room temperature. Cells were then washed with PBS, resuspended in 1 mL PBS, and analyzed by FACScan.

### 2.17 Colony assay

hNCC were pretreated with 50 μM etoposide for 48 h, sorted for senescence, and 2000 senescent cells/well were plated in 6‐well plate in duplicate with or without 500 ng/mL GH. After 10 days, colonies were stained with MTT (stock in PBS 5 mg/mL, final medium concentration 0.5 mg/mL) for 1 h at 37°C and 5% CO2, and assessed. Colony size was measured with ImageJ.

### 2.18 Cell migration assay

hNCC were treated with 50 μM etoposide for 48 h, washed, stained for live cell SA‐β‐gal expression, and sorted for SA‐β‐gal positivity. Senescent cells were plated into 75 cm flasks in full media with or without 500 ng/mL GH. Media was changed every other day. After 10 days, cells were stained for SA‐β‐gal again and sorted a second time for SA‐β‐gal–negative population. Approximately 85% of cells were SA‐β‐gal‐negative.

In total, 300,000 of these post‐senescent cells were plated into migration chambers in 100 μL of serum‐free media (with or without GH). Transwell migration chambers (Corning, cat#354578, pore size 8 M) were placed into 750 μL of full media (with or without GH) for 24 h. After staining with crystal violet, images were taken at 10× magnification (at least 10 images from each chamber), and number of migrating cells were counted.

### 2.19 Statistics

Continuous data were tested across two groups with independent or paired Student's *t* test or ANOVA and included the factor of experimental replicate where appropriate (up to four replicates performed); in some experiments, mixed model regression was used to correct for random effects across experimental replicates, and post hoc testing was performed by Tukey test to control for multiple comparisons. Residuals were inspected to confirm fit of data, and where outliers were present, data were log‐transformed prior to analysis, and are presented as mean ± SEM. Differences were considered significant where *p*‐values were < 0.05. SAS v9.4 and GraphPad v9 software were used for analysis. In experiments where data are graphed as percent of control (100%), statistical testing was performed on raw numbers.

### 2.20 Study approval

Animal experiments were approved by the Cedars‐Sinai Institutional Animal Care and Use Committee (IACUC #5587). Generation of iPSC from fibroblasts obtained from healthy human volunteer donors at Cedars‐Sinai was approved by the Cedars‐Sinai Medical Center Institutional Review Board (IRB #27264). Written informed consent was received prior to participation.

## 3 RESULTS

### 3.1 npGH is upregulated in different senescence types and is a SASP component

**FIGURE 1:**
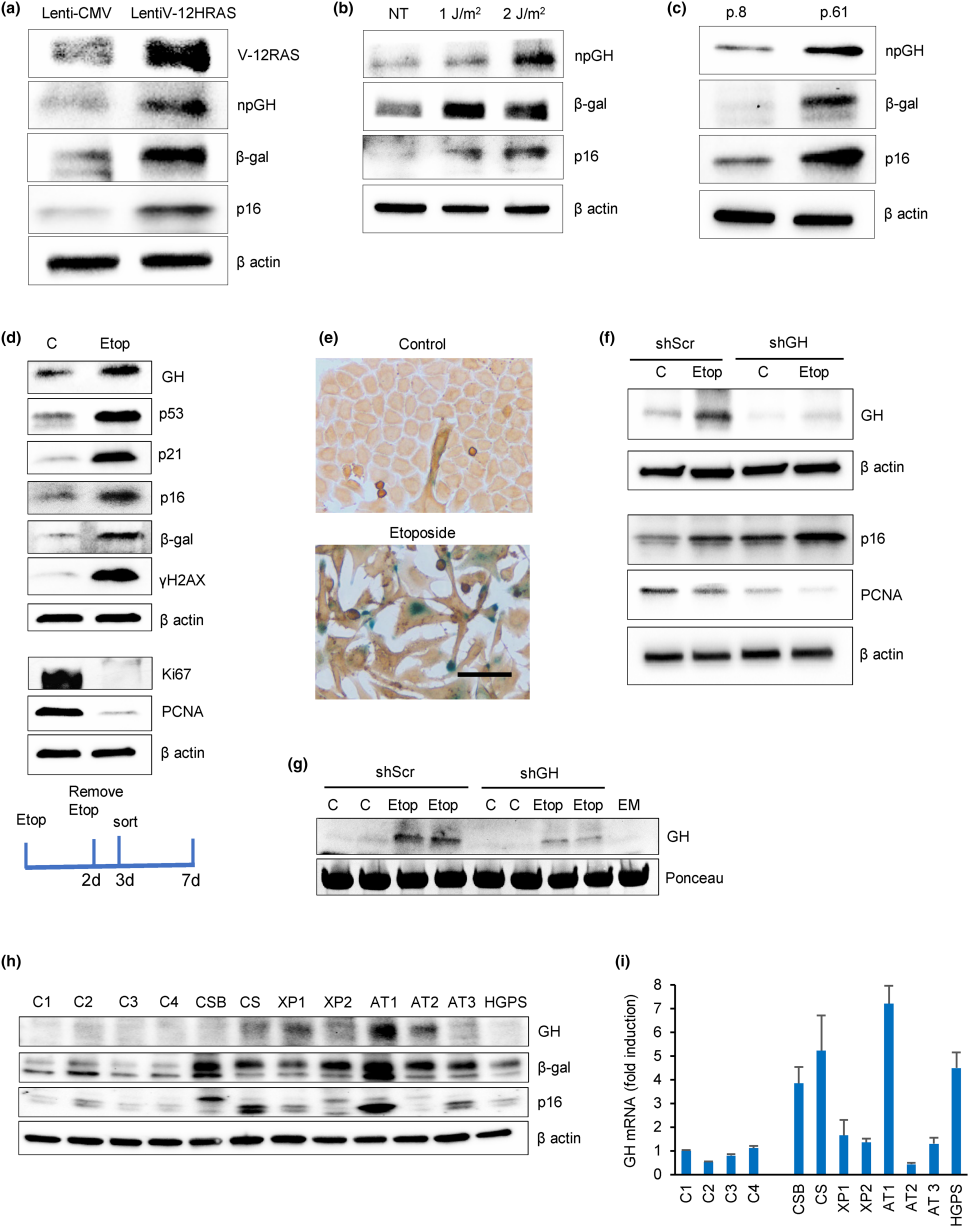
GH is induced in different senescence models and is a SASP component. (a) Oncogene‐induced senescence. Western blot of hNCC infected with lentivirus‐expressing, constitutively activated mutant V‐12HRAS oncogene (lentiV‐12HRAS) or empty vector (lenti‐CMV) and analyzed 7 days later. (b) DNA damage‐induced senescence. Western blot of hNCC exposed to indicated doses of UVC light or left untreated (NT) and analyzed 6 days later. (c) Replicative senescence. Western blot of hNCC passaged until proliferation was significantly reduced. Cells from Passage 8 (p.8) and Passage 61 (p.61) were compared. (d) Western blot of senescence markers in hNCC treated with DMSO (control) or 50 μM etoposide for 48 h and analyzed 7 days after beginning treatment. (e) Representative images of hNCC treated with DMSO (Control) or with 50 μM etoposide for 48 h and stained for SA‐β‐gal (blue) and GH (brown) 6 days after beginning treatment. Note that only senescent cells express GH. Scale bar = 20 μm. (f, g) hNCC were infected with lentivirus expressing shScr as control or shGH, treated with 50 μM etoposide (Etop) or DMSO (C) for 48 h, and analyzed 6 days after beginning treatment. (f) Western blot of cell GH and senescent marker expression. (g) Western blot of secreted GH in culture medium (in duplicate). EM, empty medium. Medium concentration was equalized according to cell number in wells. Ponceau was used as a loading control. (h) Western blot of GH and senescence markers and (i) real‐time PCR of GH in human fibroblasts derived from indicated progeroid syndrome patients and healthy volunteers as controls (C). AT, ataxia telangiectasia; CS, Cockayne syndrome; CSB, Cockayne syndrome group B; HGPS, Hutchinson–Gilford progeroid syndrome; XP, xeroderma pigmentosa. ImageJ quantification of western blots is depicted in Figure S1A and S2A,B,D,E. In a–d, f, and g, representative blots from at least three independent experiments are shown.

As we showed that endogenous GH was induced in both pituitary and non‐pituitary cells in response to senescence induced by DNA‐damaging chemotherapy (Chesnokova et al., 2013; Chesnokova, Zonis, Barrett, Kameda, et al., 2019), we tested npGH expression in other models of cellular senescence. hNCC were infected with lentivirus‐expressing, constitutively active mutant V‐12HRAS plasmid to induce oncogene‐induced senescence, then analyzed 7 days later (Figure 1a and Figure S1A). Senescence was also induced by exposing cells to 1 or 2 J/m^2^ of UVC light, which induces DNA strand breaks (Cadet et al., 2005). Cells were analyzed 6 days later (Figure 1b and Figure S1B). Replicative senescence was induced by prolonged passage of hNCC (passage 61) (Figure 1c and Figure S1C). In all three models, npGH was induced and senescence was confirmed by SA‐β‐gal enzymatic activity (Figure S1D–I). As further validation for replicative senescence, telomere length was also decreased (Figure S1J).

SA‐β‐gal activity in senescent cells correlates with β‐galactosidase expression (Kurz et al., 2000; Lee et al., 2006). In all three senescence models, levels of β‐galactosidase as well as p16, another senescence marker, were elevated, and npGH expression was induced (Figure 1a–c and Figure S1A–C).

As SASP is most robust in the context of DNA damage‐induced senescence (Coppe et al., 2008), we treated hNCC with the topoisomerase II inhibitor etoposide, which generates single‐ and double‐strand DNA breaks characteristic of chemotherapy‐induced senescence (Montecucco & Biamonti, 2007). Cells were treated with 50 μM etoposide for 48 h and senescence was confirmed by decreased proliferation as evidenced by decreased Ki67 and PCNA and senescence marker induction 7 days from the beginning of treatment (Figure 1d and Figure S2A). Immunocytochemistry analysis showed markedly induced GH in SA‐β‐gal‐positive senescent cells, while GH expression was not detected in non‐senescent cells (Figure 1e).

Similar results were also observed in hNCC stably infected with lentivirus expressing either sh Scramble (lenti‐shScr) or shGH (lenti‐shGH) RNA. In control lenti‐shScr cells, etoposide treatment significantly induced GH expression after 7 days, while considerably lower levels of npGH decreased proliferation, as assessed by lower PCNA expression and p16 induction and indicative of more senescence, was observed in shGH cells (Figure 1f and Figure S2B). Of note, senescence specifically induced GH mRNA abundance in shScr cells, while mRNA of IGF1, a downstream target of GH, was not altered (Figure S2C).

Locally secreted GH was also detected from senescent cells, with concentrations of 2 ± 0.01 pg/mL measured by ELISA in control shScr senescent cell medium, while GH was undetectable in medium derived from shGH‐treated senescent cells. Western blot confirmed markedly increased GH secretion from control senescent cells compared with cells infected with lenti‐shGH (Figure 1g and Figure S2D).

GH induction in senescent cells was also observed in fibroblasts derived from progeroid syndrome patients. Genetic progeroid syndromes are characterized by premature senescence that mimic physiological aging (Carrero et al., 2016). We therefore measured GH and other senescent markers in fibroblasts derived from eight patients with progeroid syndrome and in fibroblasts derived from four healthy individuals. GH, p16, and β‐gal proteins were induced in fibroblasts derived from progeroid patients, confirming the senescence status (Figure 1h and Figure S2E). GH mRNA levels were increased in patients with progeroid syndrome (*p* = 0.04, Figure 1i), while IGF1 mRNA levels, although increased, did not reach statistical significance (*p* = 0.08, Figure S2F,G).

These results showed GH induction and secretion in senescent colon cells and also identify GH as a SASP component. We therefore next assessed whether SASP GH exerts autocrine/paracrine effects in human colon cells.

### 3.2 GH enhances colon cell senescence exit

**FIGURE 2:**
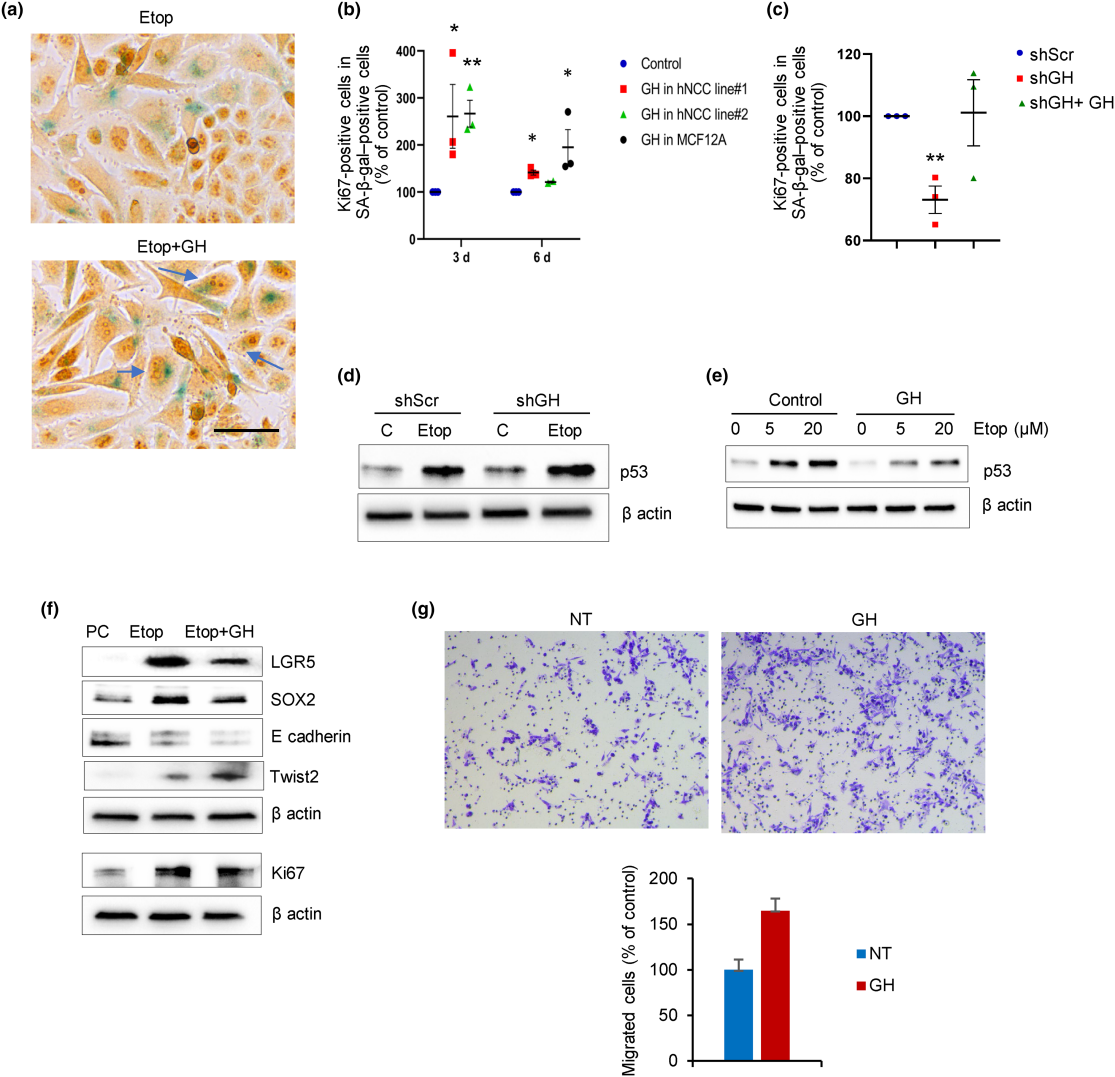
GH induces senescent cell proliferation. (a, b) hNCC were treated with 50 μM etoposide to induce senescence, treated 2 days or 5 days later with 500 ng/mL GH for 24 h, then analyzed 3 or 6 days after beginning etoposide treatment. hNCC left untreated or treated with GH only are depicted in Figure S3B. (a) Ki67 (brown) expression in senescent (blue) SA‐β‐gal–positive cells 3 days after etoposide treatment. Arrows indicate double‐positive cells. Scale bar = 20 μm. (b) Ki67‐positive cells in SA‐β‐gal‐positive cells 3 and 6 days after etoposide in hNCC line#1 and line #2, and 6 days after in MCF12A cells. (c) Ki67 in hNCC SA‐β‐gal‐positive cells transfected with lentivirus expressing either shScr (as control) or shGH and treated with 50 μM etoposide for 48 h then with 500 ng/mL GH for 24 h 5 days later. (d, e) Western blots of p53 in hNCC. (d) hNCC were stably infected with lenti‐shScr or lenti‐shGH and treated with 50 μM etoposide (Etop) or DMSO (C) for 48 h then grown for an additional 4 days.Cells were collected 6 days after begining the experiment (same as depicted in Figure 1f). Loading control is also the same as in Figure 1f. (e) hNCC were pretreated with 500 ng/mL GH overnight and then with indicated doses of etoposide (Etop) or DMSO (control) and analyzed 24 h later. (f) Western blots of hNCC treated with 50 μM etoposide, sorted for senescence 3 days later, and cultured for 14 days in the presence of GH, and sorted again, after which only SA‐β‐gal‐negative cells were analyzed. PC, parental non‐senescent cells before etoposide treatment. (g) Migration of post‐senescent hNCC. Cells were treated with etoposide and cultured in the presence of GH or not treated (NT) as in Figure 1h. 300,000 post‐senescent SA‐β‐gal‐negative cells were plated into migration chambers with or without 500 ng/mL GH and the number of migrated cells was assessed 24 h later. Graph depicts average numbers of two experiments. In b, c, each dot represents one experiment. In b and c results are graphed as a percent of control, but statistical testing was performed on raw numbers. In b, c, and, g, *p < 0.05; **p < 0.01 versus control. ImageJ quantification of western blots in d and e is depicted in Figure S4A,B,F and Figure S5A. In d, e, f, representative blots from at least three independent experiments are shown.

To examine the function of senescence‐associated npGH secretion, we recapitulated paracrine effects of GH. hNCC were treated with etoposide to induce senescence, then treated 2 days or 5 days later with 500 ng/mL GH for an additional 24 h. Thereafter, cells were analyzed 3 or 6 days after beginning the etoposide treatment. After senescence was confirmed by SA‐β‐gal activity, cells were stained for Ki67 (Figure 2a). (Non‐senescent parental hNCC left untreated or treated with GH exhibit very few senescent cells and are depicted in Figure S3A.) Senescence was also confirmed by p53 and p16 induction in these cells (Figure S3B). We assessed paracrine GH action on senescent colon cells derived from 2 different cell donors (hNCC line#1 and hNCC line#2). In SA‐β‐gal‐positive cells derived from both donors, senescent cell Ki67 expression was increased by GH at 3 (*p* < 0.01) and 6 days (*p* < 0.05) after etoposide. To examine cell‐specific capability of senescent cells to reenter the cell cycle, we employed human MCF12A cells derived from non‐tumorous breast tissue as well as human normal colon fibroblasts and human epidermal keratinocytes, all treated with etoposide and GH as described above for hNCC. Unlike rapidly proliferating colon cells, where the number of senescent cells expressing Ki67 was high, increasing from 9.75 ± 2.2% to 21.72 ± 2.06% on Day 3 and from 11 ± 1.6% to 16.1 ± 2.7% on Day 6 in hNCC line #1 (*p* < 0.01), highly differentiated slowly proliferating MCF12A cells exhibited no Ki67 expression in SA‐β‐gal‐positive cells on Day 3, concordant with prior reports (Roberson et al., 2005), and increased only from 2.1 ± 0.4% to 4.0 ± 0.4% (*p* < 0.05) by Day 6 in response to GH (Figure 2b). Senescent fibroblasts also showed low Ki67 expression (~2%) with no significant difference observed after GH treatment, and we did not observe Ki67 expression in senescent normal human epidermal keratinocytes at 3 or 6 days after etoposide treatment with or without GH (both not shown). Thus, the potential for senescent cells to reenter the cell cycle appears to be cell specific.

Consistent with these findings, BrdU incorporation also increased after GH treatment of etoposide‐treated senescent hNCC (*p* < 0.01) and in hNCC undergoing replicative senescence (Passage 61) (*p* < 0.05, Figure S2C,D), indicating that senescent cells were reentering the cell cycle.

We then infected hNCC with lentivirus‐expressing shGH, treated with etoposide as above, and stained 6 days later. Following GH knockdown, fewer senescent cells were Ki67‐positive as compared with control senescent lenti‐shScr cells (*p* < 0.01), while treatment of these cells with exogenous GH for 24 h rescued proliferation in senescent cells (Figure 2c).

We also tested whether nutlin, which induces DNA damage pathway, exerts similar effects. hNCC were treated with 3 μM nutlin3 (Vassilev et al., 2004) for 48 h to induce senescence and then with GH for an additional 24 h. Senescent cells treated with GH exhibited fourfold increased Ki67 positivity compared with cells treated with nutlin3 only (4.89 ± 1.6% vs. 23.24 ± 1.2%, *p* < 0.01). Thus, GH induces proliferation of early stage senescent cells.

To understand mechanisms of senescent cell proliferation, we assessed autonomous effects of npGH activation in senescent cells. hNCC infected with lenti‐shGH or lenti‐shScr as control were treated with etoposide to induce senescence. Etoposide was removed after 48 h, and senescent cells were further cultured for an additional 4 days. We previously showed that DNA damage induces npGH in a p53‐dependent manner, and that GH, in turn, suppresses p53 (Chesnokova et al., 2013; Chesnokova, Zonis, Barrett, Kameda, et al., 2019). Concordant with these observations, in this experiment, baseline p53 expression was lower in control shScr cells than in control shGH cells. Etoposide, by engendering DNA damage, significantly upregulates p53, and we observed more modestly upregulated p53 in lenti‐shScr senescent cells with high npGH expression, and further p53 induction in cells with suppressed npGH (Figure 2d and Figure S4A), indicating that induced npGH in senescent cells suppresses p53. Thus, induced GH may alter p53 action. Confirming paracrine npGH action, we observed that p53 was upregulated in hNCC treated with etoposide but attenuated in the presence of exogenous GH (Figure 2e and Figure S4B). Support for this hypothesis also derives from experiments with hypophysectomized rats injected with oxaliplatin. To examine effects of local GH signaling on p53, rats were hypophysectomized to eliminate effects of circulating GH. Animals were injected with 4 mg/mL oxaliplatin daily for 5 days and a group were injected with both oxaliplatin and pegvisomant, a blocker of the GH receptor. As expected, the chemotherapy drug significantly induced both GH and p53 expression in colon tissue. However, blocking local GH signaling by pegvisomant resulted in much higher p53 induction as compared to oxaliplatin alone (Figure S4C). Thus, our results would suggest that by suppressing p53 early in senescence and increasing proliferation in senescent cells as evidenced by increased Ki67 and BrdU, autocrine/paracrine npGH, at least in part, mediates senescence evasion by enabling senescent colon cells to reenter the cell cycle.

We also examined effects of paracrine npGH on colony formation in hNCC treated with 50 μM etoposide, selected for senescence 7 days after treatment by flow cytometry, and plated in the presence or absence of GH. After 10 days, GH suppressed colony formation (*p* < 0.05, Figure S4D), likely due to apoptosis of senescent cells reentering the cell cycle (Dirac & Bernards, 2003; Fielder et al., 2017) as evidenced by induced caspase 3 (Figure S4E). However, surviving cells proliferated more avidly with GH, as evidenced by 30% increase in formed colony size (*p* < 0.01, Figure S4F).

As oncogenic mutations may accumulate if senescent cells carrying unrepaired DNA damage reenter the cell cycle (Halazonetis et al., 2008), we assessed anchorage‐independent growth in senescent hNCC with induced (lenti‐shScr) or suppressed (lenti‐shGH) npGH. Cells were treated with 50 μM etoposide for 48 h, sorted for senescence after 6 days and plated in soft agar, then assessed for colony formation after 16 days. Out of 20,000 lenti‐shScr cells plated, 1.2% cells formed colonies (240 colonies), while 0.7% (140 colonies) were formed from lenti‐shGH cells.

To examine cells exiting senescence, hNCC were treated with 50 μM etoposide for 48 h, sorted for SA‐β‐gal and other senescent markers assessed on Day 7 (Figure 1d), then cultured for an additional 10 days with or without added GH. Cells were then sorted again for SA‐β‐gal expression. Surprisingly, by that time, approximately 85% of cells had become SA‐β‐gal‐negative. Only SA‐β‐gal‐negative post‐senescent cells were analyzed by western blot and compared to parental cells not exposed to etoposide. All post‐senescent cells exhibited significantly increased Ki67 and stem cell markers LGR5 and SOX2 as compared to non‐senescent parental cells. However, in cells cultured in the presence of GH, these markers were decreased; E‐cadherin was also decreased and Twist2 was upregulated compared to control post‐senescent cells without added GH (Figure 2f and Figure S5A). These results indicate that post‐senescent colon cells acquire a stem‐cell phenotype and actively proliferate, while in the presence of GH they started to differentiate, gradually triggering EMT and resulting in increased migration in post‐senescent cells (Figure 2g and Figure S5B).

These results show that induced npGH promotes senescent cell proliferation, migration, and soft agar colony formation, likely due to EMT activation, underscoring the pro‐proliferative and tumorigenic properties of GH as a component of SASP.

### 3.3 GH elicits paracrine regulation of proliferation, DNA damage, and cell transformation

**FIGURE 3:**
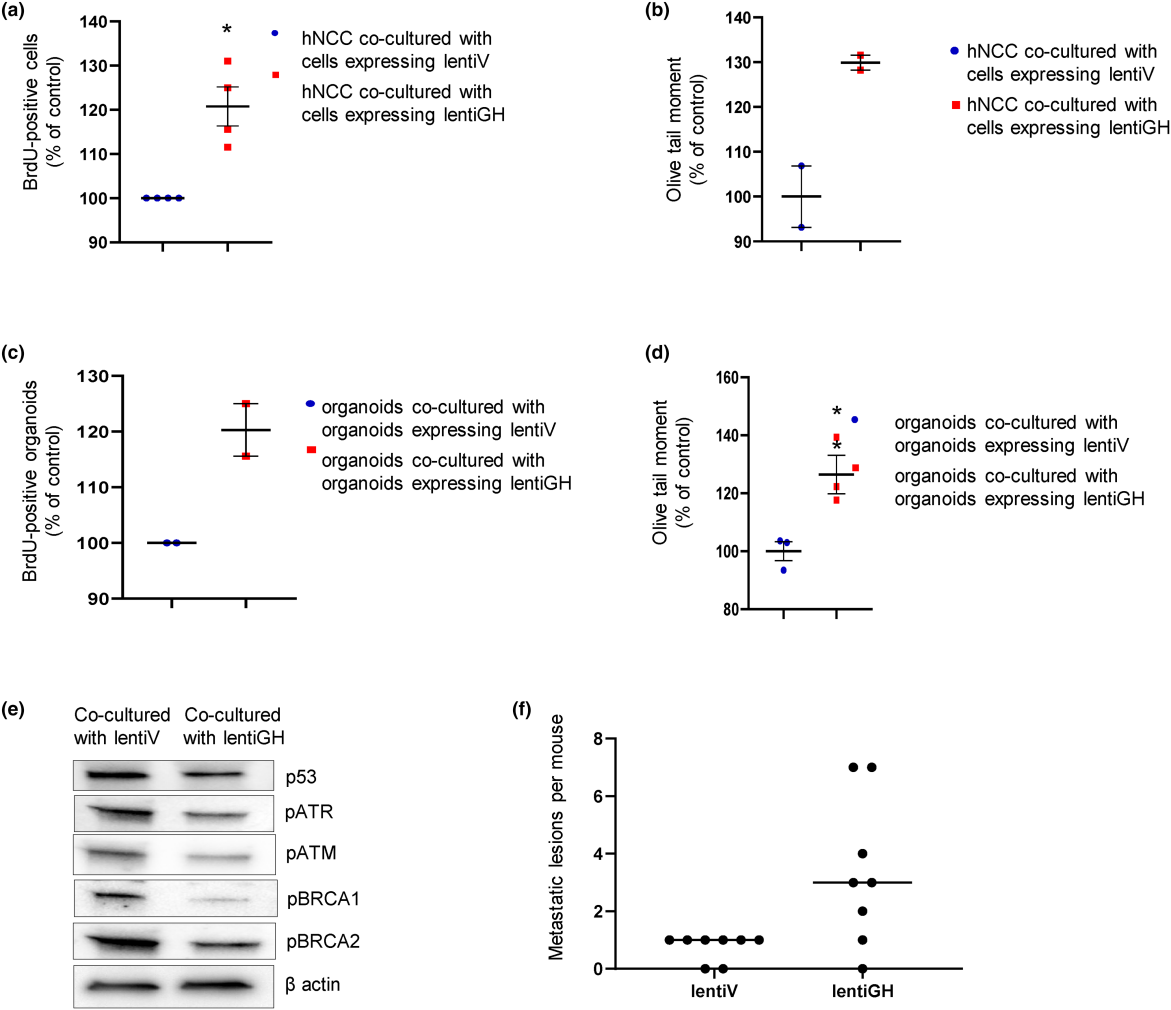
GH exerts a paracrine effect on proliferation and DNA damage. (a, b) hNCC were cocultured with hNCC infected with lentiGH or lentiV (control) for 96 h. (a) Effect on proliferation assessed by BrdU incorporation. (b) Effect on DNA damage assessed by Comet assay.(c, d) Organoids cocultured with organoids infected with lentiGH or lentiV (control) for 96 h. (c) Effect on proliferation. (d) Effect on DNA damage. Results are shown as mean ± SEM. Each dot represents one experiment. With >2 experiments, model regression was used to correct for random effects across experimental replicates, with Tukey's test to control for multiple comparisons. *p < 0.05, **p < 0.01 versus control. (e) Organoids were infected with GFP‐expressing lentiV or lentiGH and cultured for 5 weeks; organoid cells were then sorted for GFP‐positive and GFP‐negative expression. Western blot shows sorted GFP‐negative cells cocultured with lentiV or lentiGH organoid cells. Representative blots from at least three independent experiments are shown. ImageJ quantification is depicted in Figure S5B (f) Median of number of metastatic lesions developed in Nu/J mice carrying GH‐secreting (lentiGH) or control vector‐expressing (lentiV) xenografts 4 weeks after intrasplenic injection of post‐senescent hNCC, as in Figure 2f. Each dot represents one mouse.

Unrepaired DNA damage is a risk factor for tumor development (Lopez‐Otin et al., 2013; Negrini et al., 2010) and we showed that GH suppresses DNA damage repair (Chesnokova et al., 2021; Chesnokova, Zonis, Barrett, Gleeson, & Melmed, 2019; Chesnokova, Zonis, Barrett, Kameda, et al., 2019). To assess whether npGH, as a SASP component, elicits paracrine properties on neighboring cells, we cocultured untreated hNCC with hNCC infected with lentivirus expressing GH (lentiGH) or vector (lentiV) for 4 days then assessed DNA damage by comet assay and proliferation by BrdU incorporation. We observed a modest but significant 20% increase in proliferation of untreated hNCC when cocultured with lentiGH‐infected cells (*p* < 0.05, Figure 3a), as well as a trend for more abundant DNA damage in untreated hNCC cocultured with lentiGH‐ versus lentiV‐infected cells with ~30% increase calculated as a mean of two experiments (Figure 3b).

We confirmed these results in three‐dimensional intestinal organoids generated from iPSCs derived from normal human fibroblasts (Barrett et al., 2014; Workman et al., 2018) cocultured with lentiGH‐ or lentiV‐infected organoids. Similar to observations with hNCC, organoid proliferation increased by 20% upon coculture with lentiGH‐infected organoids, indicating a similar trend as observed in hNCC as a mean of two experiments (Figure 3c), and 38% more DNA damage was observed with lentiGH coculture (*p* < 0.01, Figure 3d).

To assess mechanisms underlying increased unrepaired DNA damage in lentiGH cocultures, organoids were infected with lentiV or lentiGH, both expressing GFP, and cultured for 5 weeks. We observed that approximately 60% of organoid cells were positive for GFP. Organoids were then dispersed to single‐cell suspension and sorted for GFP‐positive (expressing either lentiGH or lentiV) and GFP‐negative intact neighboring cells. In testing how paracrine GH emanating from infected cells would affect intact neighboring cells, GFP‐negative cells cocultured with GFP‐positive lentiV or lentiGH organoid cells were compared, and we found decreased DNA damage and repair proteins in organoid cells growing in close proximity with cells expressing and secreting GH (Figure 3e and Figure S5B). Thus, paracrine npGH induces proliferation, and, by suppressing DNA repair pathways, also enhances unrepaired DNA damage accumulation in neighboring cells, which may lead to cell transformation.

To assess GH effects on transformative and metastatic properties of post‐senescent cells in vivo, immunocompromised Nu/J mice were first injected subcutaneously with HCT116 cells infected either with lenti‐hGH or lentiV as control. After 3 weeks, animals developed GH‐ or vector‐expressing subcutaneous tumors. hNCC were treated with 50 μM etoposide for 48 h then sorted for senescence, and 90% were positive for SA‐β‐gal. Similar treatment resulted in induction of multiple senescence markers and almost complete downregulation of proliferation markers indicating cell cycle exit 7 days later (Figure 1d). SA‐β‐gal–positive cells were then cultured for an additional 30 days to propagate post‐senescent cells. During this time, most of these cells lost SA‐β‐gal positivity, and 2 × 10^6^ post‐senescent SA‐β‐gal–negative cells were injected intrasplenic. Animals were sacrificed 3 weeks after injection and metastases assessed. Mice bearing HCT116 lenti‐hGH tumors had high circulating hGH (2332 ± 712 ng/mL) as well as increased circulating mIGF1 (942.7 ± 64.3 ng/mL vs. 553.5 ± 41.3 ng/mL for lentiV). These animals developed fourfold more metastases versus control animals (Figure 3f) with the average size of metastatic lesions ~2× larger in this group (Table S1).

Thus, by suppressing DNA repair and inducing EMT, paracrine GH creates a tissue microenvironment favoring senescence evasion and post‐senescent cell transformation.

### 3.4 GH is induced by CXCL1 in senescent cells

**FIGURE 4:**
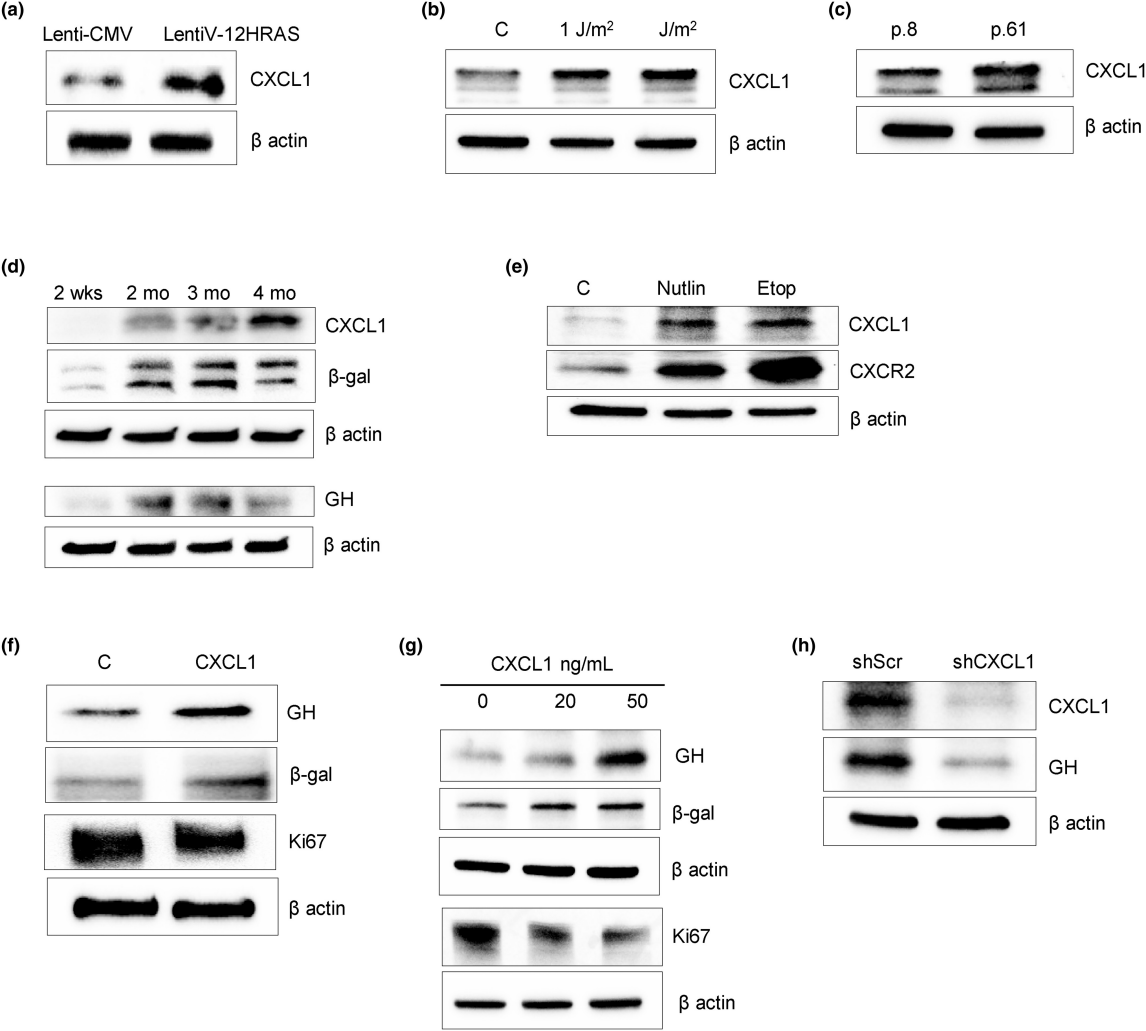
CXCL1 is expressed in senescent cells and upregulates GH. (a–e) Western blots of CXCL1 expression after senescence induction. (a) Oncogene‐induced senescence. hNCC were infected with lentivirus expressing constitutively activated HRAS oncogene (lentiV‐12HRAS) or empty vector (lenti‐CMV) and analyzed 7 days later. (b) DNA damage‐induced senescence. hNCC were exposed to indicated doses of UVC light or left untreated (C) and analyzed 6 days later. (c) Replicative senescence. hNCC were passaged until proliferation was significantly reduced. Cells from Passage 8 (p.8) and Passage 61 (p.61) were compared. In a–c, loading control is the same as in Figure 1a–c. (d) Western blot of senescent organoids cultured for up to 4 months. (e) Western blot of CXCL1 and its receptor CXCR2 in hNCC 72 h after treatment with 3 μM nutlin3 (Nutlin), 5 μM etoposide (Etop), or DMSO as control (C) for 48 h. ImageJ quantification of western blots is depicted in Figure S6A–F. (f–h) Western blots of (f) organoids treated with 100 ng/mL CXCL1 for 4 days; (g) hNCC treated with indicated doses of CXCL1 for 4 days; and (h) hNCC in replicative senescence (p.61) infected with lenti‐shCXCL1 or lenti‐shScr and analyzed 3 days after infection. ImageJ quantification of western blots is depicted in Figure S7A–D. Representative blots from at least three independent experiments are shown.

We next sought to elucidate how npGH expression is sustainably induced in senescent cells. CXCL1, as a SASP component, is expressed and secreted from senescent cells, and promotes senescence in both an autocrine and paracrine manner (Gorgoulis et al., 2019). We detected upregulated CXCL1 in lentiV12 HRAS‐infected oncogene‐induced senescent hNCC, in UVC‐exposed irradiated cells, and in replicative senescent cells (Figure 4a–c and Figure S6A–C). To test the association between CXCL1 and npGH in stably senescent cells, we cultured intestinal organoids for up to 4 months, at which point they had exhausted their proliferative potential and achieved replicative senescence. SA‐β‐gal activity (Figure S6D) and senescence markers β‐galactosidase and CXCL1 were induced, as was GH expression (Figure 4d and Figure S6E).

We also observed that CXCL1 and its receptor CXCR2 were markedly upregulated in chemotherapy‐induced senescent hNCC (Figure 4e and Figure S6F) treated with etoposide or with nutlin3.

As CXCL1 is secreted, we treated organoids for 4 days with 100 ng/mL CXCL1 and observed β‐galactosidase induction, decreased Ki67, and markedly increased npGH (Figure 4f and Figure S7A). Accordingly, npGH and IGF1 mRNA levels were also upregulated after CXCL1 treatment, while prolactin mRNA did not change (Figure S7B). Treatment of hNCC for 4 days with CXCL1 produced similar results. CXCL1 dose‐dependently suppressed Ki67, while β‐galactosidase was upregulated, and npGH expression also increased dose‐dependently (Figure 4g and Figure S7C). By contrast, hNCC infected with lentivirus expressing shCXCL1 had suppressed npGH (Figure 4h and Figure S7D), supporting our hypothesis that CXCL1 secreted by senescent cells sustains upregulated local npGH.

### 3.5 GH suppresses CXCL1 expression in senescent cells by inhibiting NFκB activity

**FIGURE 5:**
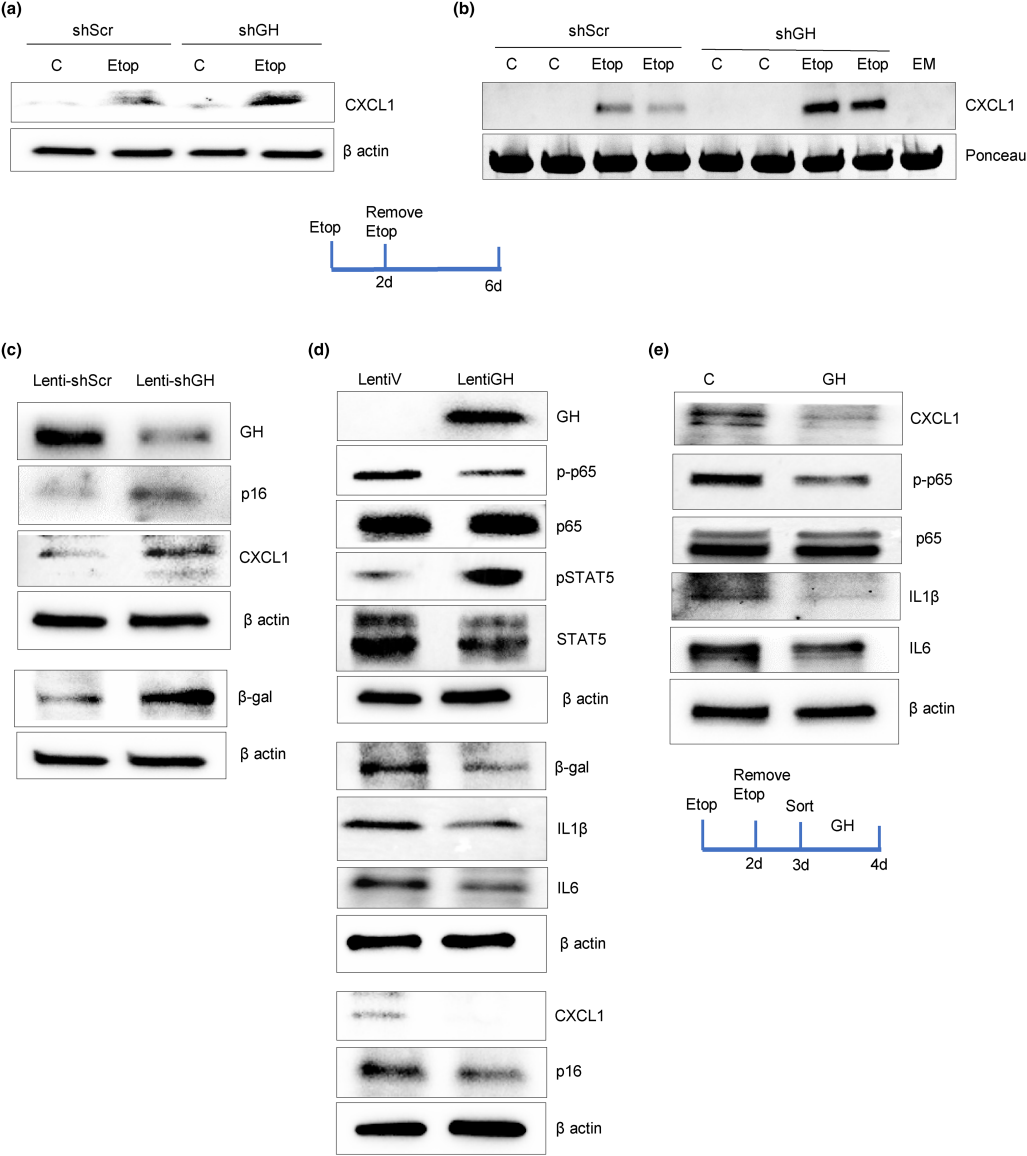
Senescent cell GH suppresses CXCL1 by inhibiting NFκB activity, and changes cytokine SASP content. (a, b) hNCC were infected with either lenti‐shGH or lenti‐shScr, treated with 50 μM etoposide (Etop) to induce senescence or with DMSO as control (C) then analyzed 6 days later. Western blot of CXCL1 in (a) cells and (b) culture medium (in duplicate). EM, empty medium. (c, d) Western blot of organoids 1 week after infection with (c) lenti‐shGH or lenti‐shScr and (d) lentiGH or lentiV. (e) Western blot of hNCC treated with 50 μM etoposide for 2 days, sorted for senescence on Day 3, and treated with GH for an additional 24 h. Representative blots from at least three independent experiments are shown. ImageJ quantification of western blots is depicted in Figure S8. Schematic representations depict experimental design.

As we observed that feedback between CXCL1 and GH in senescent cells also enables induced npGH to suppress CXCL1, hNCC infected with lenti‐Scr and lenti‐shGH were treated with DNA‐damaging etoposide for 48 h. After 6 days, we observed that both CXCL1 expression (Figure 5a and Figure S8A) and secretion (Figure 5b and Figure S8B) were increased with suppressed npGH. Similar results were obtained using real‐time PCR showing considerably higher levels of CXCL1 mRNA in senescent cells with attenuated GH (Figure S8C).

Suppressing npGH in organoids cultured for 2 months with lenti‐shGH induced CXCL1, β‐gal, and p16 after 1 week, reflective of a senescent state (Figure 5c and Figure S8D). By contrast, organoids infected with lentiGH exhibited reduced β‐gal, p16, and CXCL1 (Figure 5d and Figure S8E).

NFκB, a transcription factor for CXCL1 expression (Burke et al., 2014), requires phosphorylated p65 (RelA) as a transcriptional regulator (Oeckinghaus & Ghosh, 2009). We found that GH suppressed organoid p65 phosphorylation, thereby constraining NFκB transcriptional activity. As activated STAT5 reduces NFκB activity (Gilbert et al., 2012) and GH signals to phosphorylate STAT5 (Andrews et al., 2001; Waters, 2016), it is likely that GH regulates p65 phosphorylation by activating STAT5, as evidenced in organoids expressing high levels of GH (Figure 5d and Figure S8E). Experiments in senescent hNCC confirmed this observation. Cells were treated with 50 μM etoposide for 48 h, sorted for senescence on Day 3, and treated with 500 ng/mL GH for an additional 24 h. Both phosphorylated p65 and CXCL1 were downregulated in GH‐treated cells. Moreover, other SASP components, including IL1β and IL6, were also suppressed both in organoids (Figure 5d and Figure S8E) and hNCC (Figure 5e and Figure S8F), likely due to NFκB suppression.

Thus, in senescent colon cells, CXCL1 induces npGH, which, in turn, attenuates CXCL1 expression and secretion and modulates SASP content.

### 3.6 GH suppresses colon CXCL1 in vivo

**FIGURE 6:**
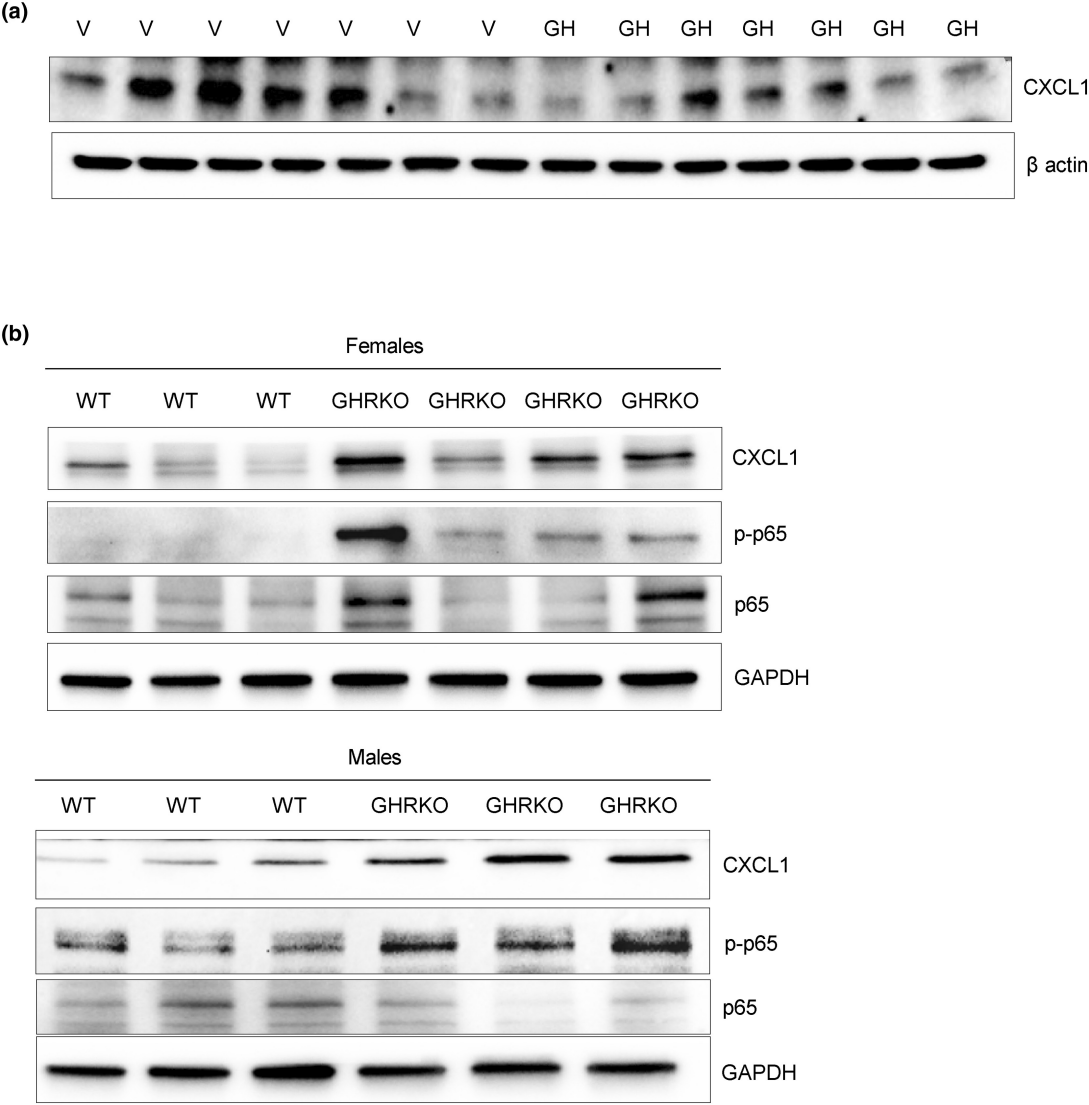
GH suppresses CXCL1 in vivo. (a) Western blot of CXCL1 in colon tissue from female nude mice implanted with HCT116 GH‐secreting xenografts (GH) or empty vector (V) and sacrificed 5 weeks later. (b) GH signaling deficiency results in CXCL1 upregulation in vivo. Western blot of CXCL1, p‐p65, and p65 in colon tissue of female and male GHR knockout (GHRKO) and wild‐type (WT) mice. ImageJ quantification of western blots is depicted in Figure S9.

To affirm these results in vivo as a proof of concept, we injected athymic female nude mice subcutaneously with human colon adenocarcinoma HCT116 cells stably expressing mGH or empty vector. All HCT116 xenografts formed tumors that secreted abundant GH in experimental animals, achieving ~10‐fold increased circulating GH levels as shown (Chesnokova et al., 2016). Five weeks after implantation, mice were sacrificed, and colon CXCL1 expression was markedly attenuated in mice implanted with GH‐secreting xenografts (Figure 6a and Figure S9A).

By contrast, male and female GHRKO mice devoid of GH signaling had increased levels of colon CXCL1 as well as phosphorylated p65, consistent with activated CXCL1 (Figure 6b and Figure S9B). These in vivo findings buttress our hypothesis that GH suppresses CXCL1 expression while GH deficiency leads to CXCL1 upregulation.

## 4 DISCUSSION

We show here that colon npGH as a SASP component is induced in response to three different types of cellular senescence, that SASP npGH increases senescence evasion and cell transformation, and that npGH induction in senescent cells is supported by the SASP chemokine CXCL1. These results highlight a novel signaling function for GH.

Longevity depends on multiple factors (Longo & Anderson, 2022). Cell senescence and SASP composition depend both on tissue type and the nature of senescence‐inducing stimuli (Basisty et al., 2020; Hernandez‐Segura et al., 2017). We have shown that GH suppresses p53, increases normal colon cell motility and transformation, and enhances anchorage‐independent colony formation (Chesnokova et al., 2016; Chesnokova, Zonis, Barrett, Kameda, et al., 2019). Here, we show that SASP‐derived GH exhibits similar autonomous effects. npGH is induced and secreted from senescent colon cells, stimulating proliferation, while senescent cells with suppressed GH form fewer soft agar colonies than do control senescent cells with intact GH expression. Furthermore, p53 levels were higher in senescent hNCC with suppressed endogenous GH, concordant with known GH suppression of p53. p53 restrains cell cycle progression, and is required for senescence induction and support. As suppressing p53 can still reverse early stages of senescence (Beausejour et al., 2003; Lee & Schmitt, 2019), GH‐dependent increased proliferation may occur as a result of p53 suppression.

These results demonstrate a heretofore unappreciated npGH action, whereby npGH induced in non‐transformed senescent epithelial cells leads to senescence evasion, thus enabling cell cycle reentry.

Our results seemingly are not consistent with reports from the Kirkland group where GH activity was positively associated with an increased senescent burden in white adipose tissue, while in GH‐deficient or resistant animals this effect was attenuated (Stout et al., 2014). As GH suppresses DNA damage repair, the resulting DNA damage could enhance senescent cell abundance, while in GH‐deficient mice DNA repair is more efficient. Our earlier finding that *GHR* ^*−/−*^ mice exhibit less colon DNA damage supports this mechanism (Chesnokova et al., 2021).

Although senescence is defined by irreversible growth arrest, several studies have demonstrated reversibility of the senescent state in non‐tumorous cells (Ei et al., 2021; Martin et al., 2014; Romanov et al., 2001) and in cancer cells after chemotherapy (Gosselin et al., 2009; Saleh et al., 2019). In the different cellular senescent models we studied, local npGH induction was associated with both p53 suppression and increased proliferation. Senescent cells reentering the cell cycle when p53 is downregulated may be eliminated by apoptosis, while some continue to proliferate (Beausejour et al., 2003; Dirac & Bernards, 2003; Fielder et al., 2017). Tumor cells evading chemotherapy‐induced senescence may become aggressive and chemo‐resistant (Milanovic et al., 2018; Saleh et al., 2018). Senescence evasion in precancerous cells may also lead to benign‐to‐neoplastic progression (Haugstetter et al., 2010; Lee & Schmitt, 2019; Michaloglou et al., 2005; Yu et al., 2018). As DNA damage is abundant in senescent cells (Fumagalli et al., 2014), by reentering the cell cycle, they may be vulnerable to acquiring oncogenic mutations (Aunan et al., 2017; Petr et al., 2020). Our in vivo experiments affirmed this hypothesis, as mice with high circulating GH injected with post‐senescent cells developed more metastases than did control animals with normal circulating GH. Of note, although we confirmed senescence 7 days after etoposide treatment by the induction of multiple senescent markers and a striking decrease in proliferation markers, we cannot exclude that some SA‐β‐gal–positive cells may never become fully senescent and may continue to proliferate during long‐term culturing; this may resemble effects of chemotherapy in tumors and neighboring non‐tumorous cells. Our experiments performed on non‐transformed human colon cells and organoids show that, by suppressing p53, induced GH may intensify senescence evasion, whereby proliferating senescent cells, unlike parental cells cultured for the same duration, undergo transformation, as evidenced by enhanced upregulation of EMT markers and anchorage‐independent growth mediated by GH.

Senescence evasion occurs as a cell‐specific event, and while senescent colon cells treated with GH readily escape and start to proliferate, only a small percentage of senescent normal breast cells and colon fibroblasts become Ki67‐positive after GH treatment. Indeed, here we show dual effects of SASP GH, whereby the hormone attenuates senescent cell colony formation, likely by inducing apoptosis, while triggering surviving senescence cell proliferation and transformation.

Colon cell sensitivity to GH may underlie increased colon polyp and colon adenocarcinoma seen in acromegaly patients (Gonzalez et al., 2017) and with age (Maratt et al., 2017).

Non‐autonomous effects of SASP‐derived npGH should also be considered. As GH suppresses DNA damage repair (Chesnokova, Zonis, Barrett, Kameda, et al., 2019), SASP‐associated npGH may also promote DNA damage accumulation in neighboring cells. Given that GH‐overexpressing hNCC and organoids elicit a paracrine effect on cocultured untreated hNCC and organoids, respectively, increasing both proliferation and DNA damage, we interpret these results as indicating that suppressed DNA repair pathways evident in organoid cells cocultured with cells expressing and secreting paracrine GH underlie DNA damage accumulation. These observations further highlight mechanisms for pro‐proliferative and pro‐neoplastic SASP properties. Our results suggest that GH, as a component of SASP and acting in an autocrine/paracrine manner, may enable a pro‐neoplastic environment by accumulation of damaged DNA in non‐tumorous cells.

We show here that induction of SASP‐associated GH requires CXCL1, known to induce fibroblast senescence (Kim et al., 2018; Yang et al., 2006). Microenvironmental chemokines communicate between senescent and non‐senescent cells, and promote inflammation and tumorigenesis. CXCL1, as a SASP component, functions through both autocrine and paracrine pathways (Lee et al., 1992; Loukinova et al., 2000). It acts in a tissue‐ and cell‐specific manner and is induced during oncogene‐induced senescence (Acosta & Gil, 2009; Lesina et al., 2016), during fibroblast replicative senescence, and in preneoplastic prostate lesions (Acosta et al., 2013), and also attracts neutrophils to eliminate senescent cells (Kim et al., 2018; Yang et al., 2006). CXCL1 binds the CXCR2 receptor that also binds IL8, CXCL2, and CXCL5 (Lee et al., 1992), further reinforcing senescence (Acosta et al., 2008). It is likely that, by inducing senescence, CXCL1 activates p53, which, in turn, binds the GH promoter to enhance its expression (Chesnokova et al., 2016).

We observed universal CXCL1 induction in different models of non‐tumorous colon cell senescence. In turn, high levels of CXCL1 activated senescence markers, notably p16, while Ki67 was suppressed, indicative of decreased proliferation. CXCL1 also induces npGH, thereby driving cells to evade senescence and reenter the cell cycle. Surprisingly, we observed that upregulated GH, in turn, suppresses CXCL1 expression, likely enabling a positive–negative regulatory loop for CXCL1 production. As chronic GH administration reduces NFκB (Han et al., 2007), and exogenous GH suppressed organoid p65 phosphorylation, inactivating NFκB effects on CXCL1 are consistent with known anti‐inflammatory GH properties (Masternak & Bartke, 2012; Soendergaard et al., 2017). Taken together, our results suggest that by inhibiting NFκB activity, GH downregulates SASP IL1β and IL6. Results of in vivo experiments with high levels of circulating GH further affirm the suppressive GH effects on CXCL1. By contrast, GHRKO mice devoid of GH signaling exhibit increased colon phospho‐NFκB associated with elevated CXCL1. Thus, npGH modulates SASP composition by inhibiting CXCL1. Although CXCL1 is a pro‐senescence factor, the molecule also contributes to phagocytic elimination of senescent cells (Binet et al., 2020; Calcinotto et al., 2019). Thus, CXCL1 suppression by npGH in non‐tumorous senescent cells may protect senescent cells from elimination and enable senescence evasion. Of note, tumor cell SASP suppression may also account for chemotherapy resistance, as natural killer cells are attracted by SASP components (Ruscetti et al., 2020). As GH is induced in response to DNA damaging therapy, this mechanism may, at least in part, underlie known GH/GHR‐mediated chemotherapy resistance (Basu & Kopchick, 2019). Our results thus also shed light on as yet insufficiently explored mechanisms underlying changes in neighboring non‐tumorous epithelial cells after DNA damaging therapy.

We previously showed that senescent breast cancer MCF7 and colon cancer HCT116 cells also secrete npGH (Chesnokova et al., 2013). The results shown here support npGH contribution to the complexity of SASP composition and actions. npGH may at least partially contribute to pro‐proliferative SASP properties, and as DNA damage accumulation is characteristic of senescence pathways and is also a hallmark of aging (Lopez‐Otin et al., 2013), npGH enhancement of DNA damage in non‐tumorous neighboring cells may contribute to age‐associated tissue degeneration and promotion of neoplasia. This postulate is exemplified by reports that endometrial, breast, liver, prostate, and colon cancers all express endogenous npGH (Perry et al., 2008, 2017). Notably, intratumoral GH abundance in hepatocellular, uterine, and breast cancer correlates highly with disease progression, and is an adverse mortality determinant (Kong et al., 2016; Perry et al., 2017; Wu et al., 2011).

In light of the increased murine lifespan associated with GH deficiency (Bartke, 2020; Colon et al., 2019; Young et al., 2021) as well as the observed cancer absence in patients harboring a *GHR* mutation that disrupts GH signaling (Guevara‐Aguirre et al., 2011; Johannsson & Kopchick, 2021), these results suggest that induced SASP npGH may constitute an adverse signal contributing to age‐associated diseases (Kopchick et al., 2022). These results also highlight the risk of inappropriate use of GH as an antiaging (Giordano et al., 2008; Medeiros & Siegel Watkins, 2018; Melmed, 2019) or as a performance enhancing drug for athletes (Holt & Ho, 2019; Liu et al., 2008).

Overall, the results presented here offer novel insights for understanding the role of GHR signaling in mediating heretofore unknown GH actions regulating the adult epithelial microenvironment.

## AUTHOR CONTRIBUTIONS

VC and SM developed the hypothesis and wrote the manuscript. VC, SZ, TA, and RB conducted experiments. VC and SM analyzed, discussed, and interpreted the data. SM coordinated and directed the project. All authors approved the submitted manuscript.

## CONFLICT OF INTEREST STATEMENT

The authors declare no competing interests.

## Supporting information


Appendix S1.

## Data Availability

The data that support the findings of this study are available in this article and/or the supplementary material of this article.
