# Supplementary material for: Non‐pituitary growth hormone enables colon cell senescence evasion
Source: Aging Cell. 2024 May 9;23(8):e14193. doi: 10.1111/acel.14193 (PMC11320355; doi:10.1111/acel.14193)
Supplement: Supplementary file 1 — Appendix S1. [file ACEL-23-e14193-s001.pdf]

Non-pituitary growth hormone enables colon cell senescence evasion

Vera Chesnokova, Svetlana Zonis, Tugce Apaydin, Robert Barrett, Shlomo Melmed

Supplemental Information

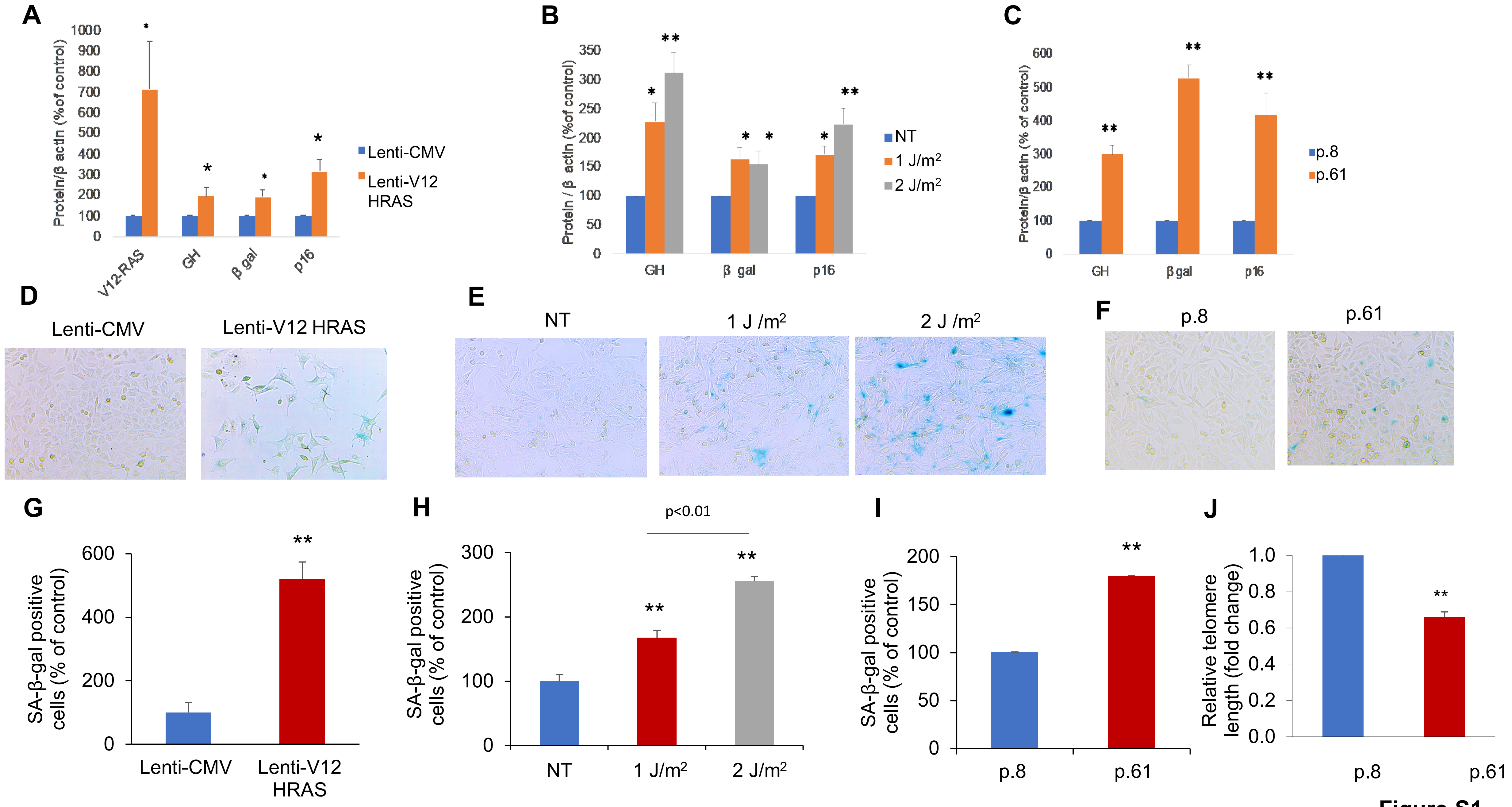
A
Protein/β actin (%of control)
Lenti-CMV
Lenti-V12 HRAS
V12-RAS
GH
β gal
p16
B
Protein / β actin (%of control)
NT
1 J/m2
2 J/m2
C
Protein/β actin (% of control)
p.8
p.61
D
Lenti-CMV
Lenti-V12 HRAS
E
NT
1 J /m2
2 J /m2
F
p.8
p.61
G
SA-β-gal positive cells (% of control)
Lenti-CMV
Lenti-V12 HRAS
H
p<0.01
SA-β-gal positive cells (% of control)
NT
1 J/m2
2 J/m2
I
SA-β-gal positive cells (% of control)
p.8
p.61
J
Relative telomere length (fold change)
p.8
p.61

Figure S1

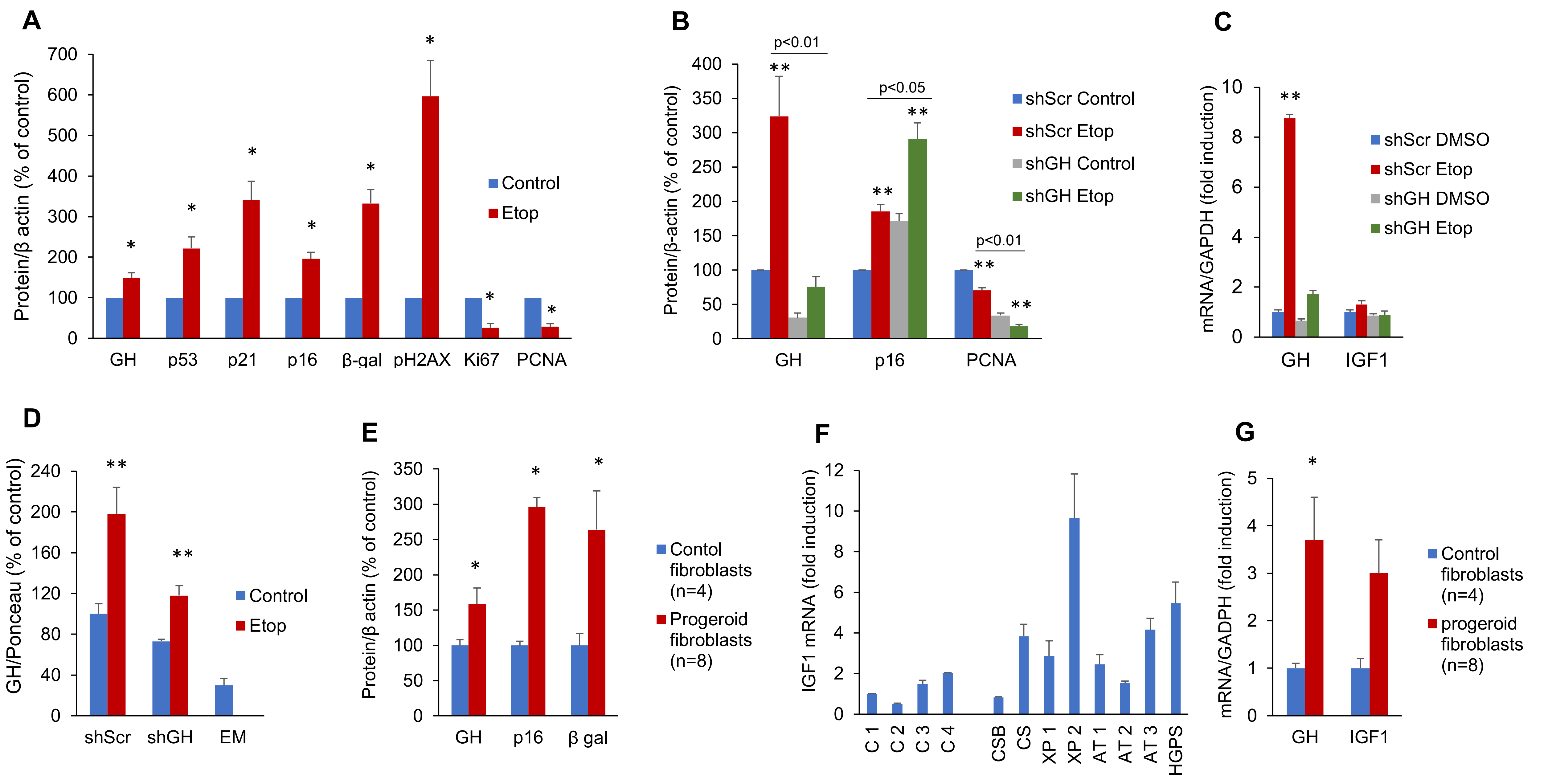
A
Protein/β actin (% of control)
Control
Etop
GH
p53
p21
p16
β-gal
pH2AX
Ki67
PCNA
B
Protein/β-actin (% of control)
p<0.01
p<0.05
p<0.01
shScr Control
shScr Etop
shGH Control
shGH Etop
GH
p16
PCNA
C
mRNA/GAPDH (fold induction)
shScr DMSO
shScr Etop
shGH DMSO
shGH Etop
GH
IGF1
D
GH/Ponceau (% of control)
Control
Etop
shScr
shGH
EM
E
Protein/β actin (% of control)
Contol fibroblasts (n=4)
Progeroid fibroblasts (n=8)
GH
p16
β gal
F
IGF1 mRNA (fold induction)
C 1
C 2
C 3
C 4
CSB
CS
XP 1
XP 2
AT 1
AT 2
AT 3
HGPS
G
mRNA/GADPH (fold induction)
Control fibroblasts (n=4)
progeroid fibroblasts (n=8)
GH
IGF1

Figure S2

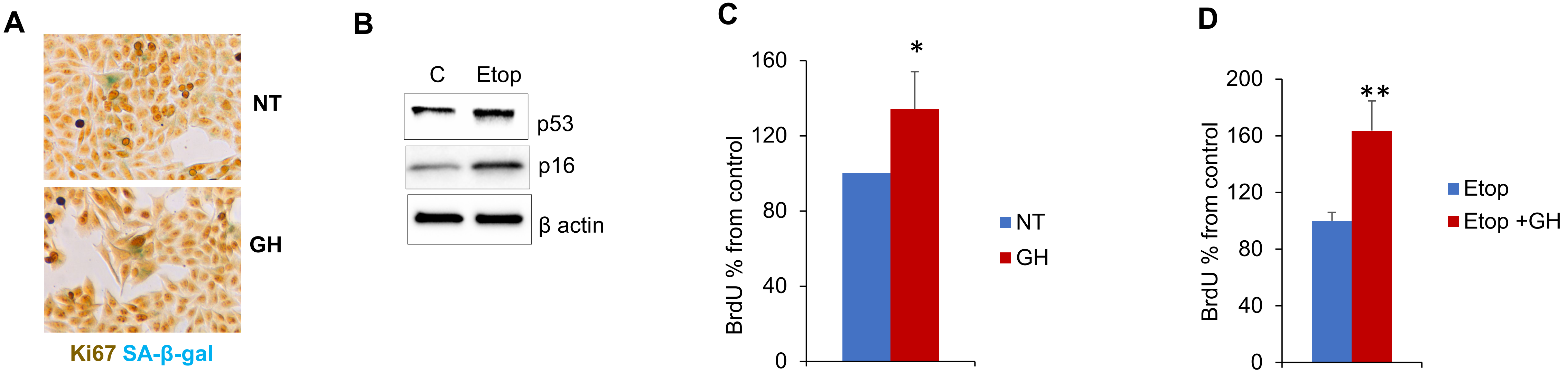
A
NT
GH
Ki67 SA-β-gal
B
C
Etop
p53
p16
β actin
C
BrdU % from control
160
120
80
40
0
*
NT
GH
D
BrdU % from control
200
160
120
80
40
0
**
Etop
Etop +GH

Figure S3

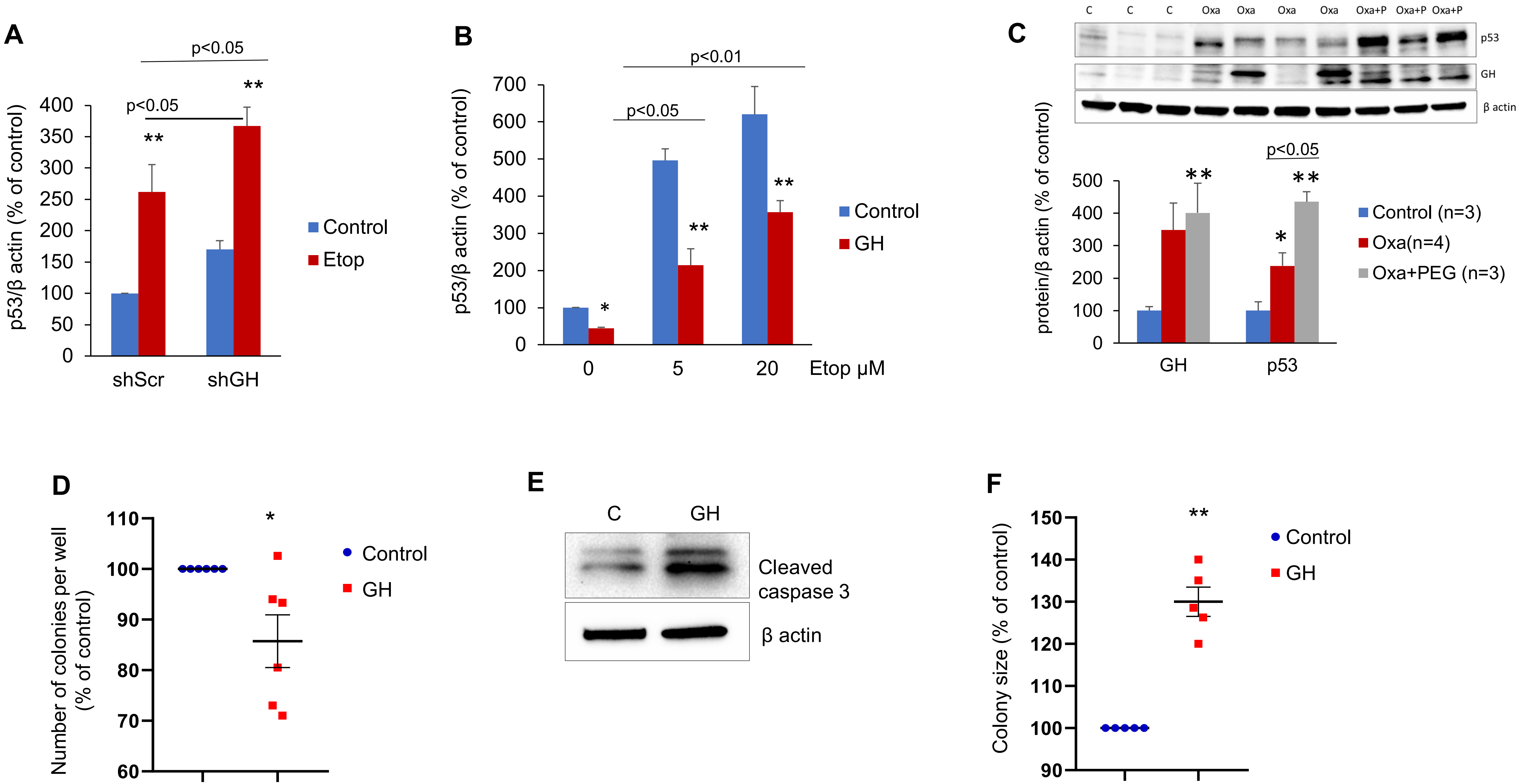

A
p<0.05
p<0.05
**
**
p53/β actin (% of control)
400
350
300
250
200
150
100
50
0
shScr
shGH
Control
Etop
B
p<0.01
p<0.05
*
**
**
p53/β actin (% of control)
700
600
500
400
300
200
100
0
0
5
20
Etop µM
Control
GH
C
C C C Oxa Oxa Oxa Oxa Oxa+P Oxa+P Oxa+P
p53
GH
β actin
p<0.05
**
*
**
protein/β actin (% of control)
500
400
300
200
100
0
GH
p53
Control (n=3)
Oxa(n=4)
Oxa+PEG (n=3)
D
*
Number of colonies per well (% of control)
110
100
90
80
70
60
Control
GH
E
C
GH
Cleaved caspase 3
β actin
F
**
Colony size (% of control)
150
140
130
120
110
100
90
Control
GH

Figure S4

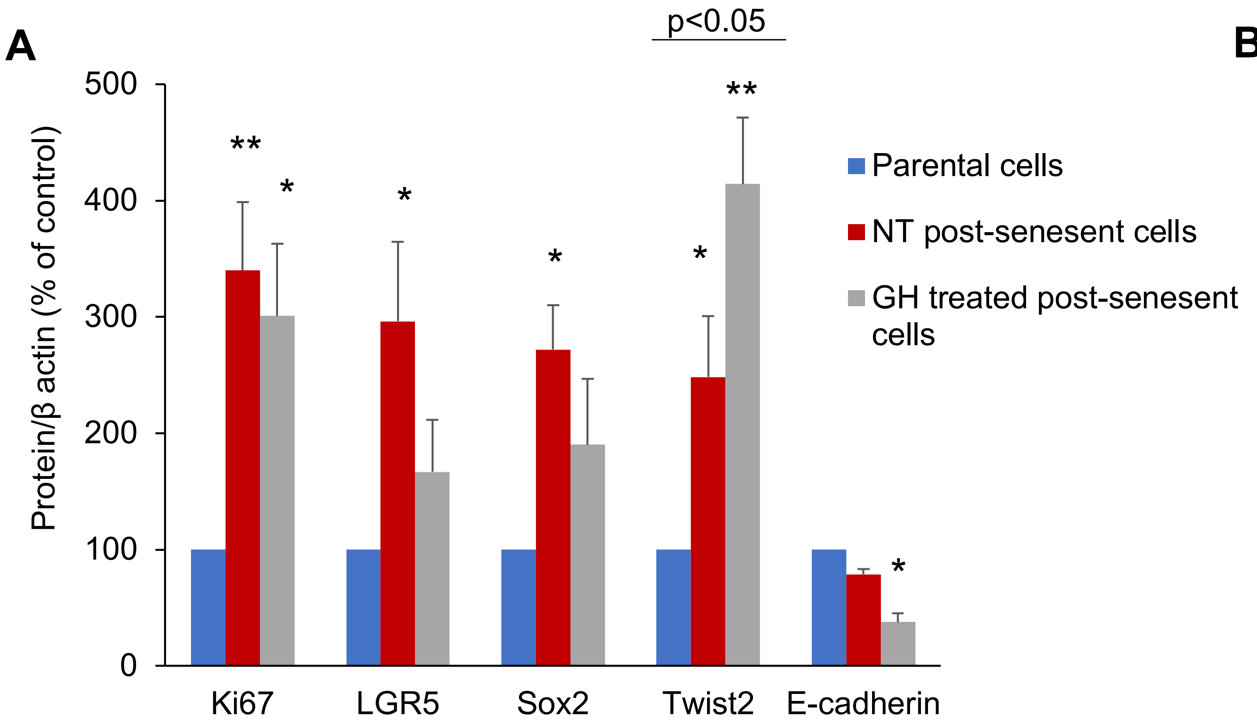
A
p<0.05
Protein/β actin (% of control)
500
400
300
200
100
0
** * * * * **
*
Ki67
LGR5
Sox2
Twist2
E-cadherin
Parental cells
NT post-senesent cells
GH treated post-senesent cells

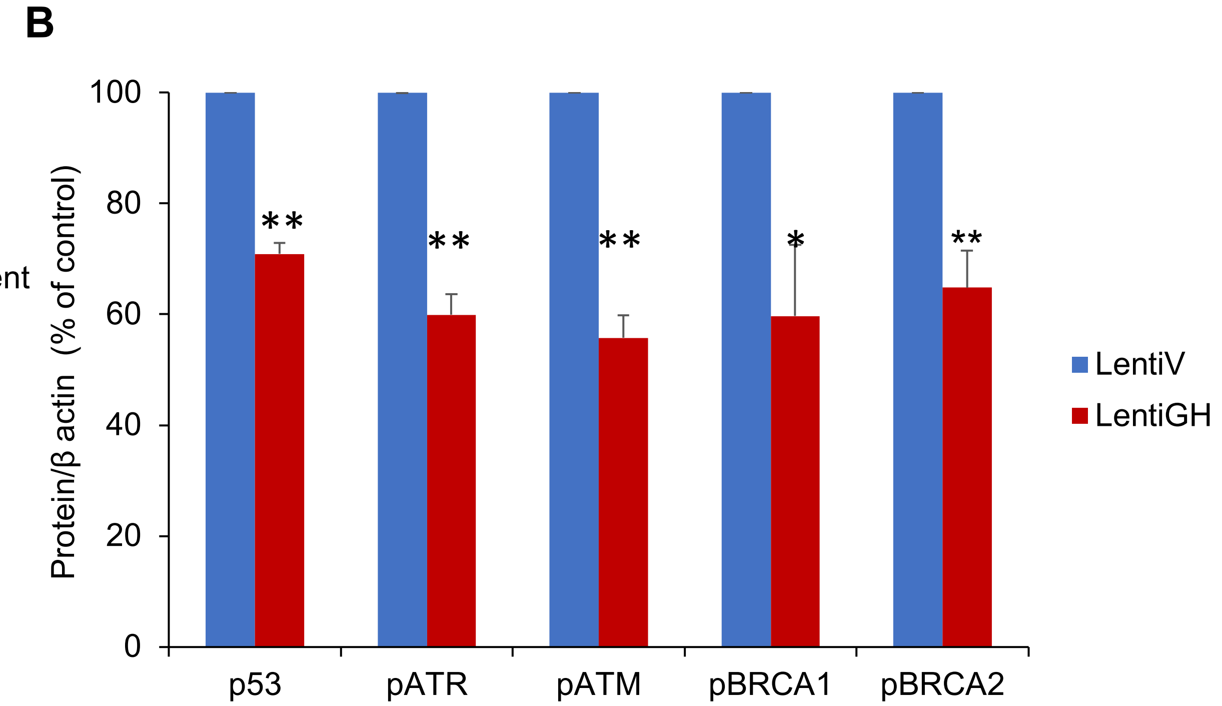
B
Protein/β actin (% of control)
100
80
60
40
20
0
** ** ** * **
p53
pATR
pATM
pBRCA1
pBRCA2
LentiV
LentiGH

Figure S5

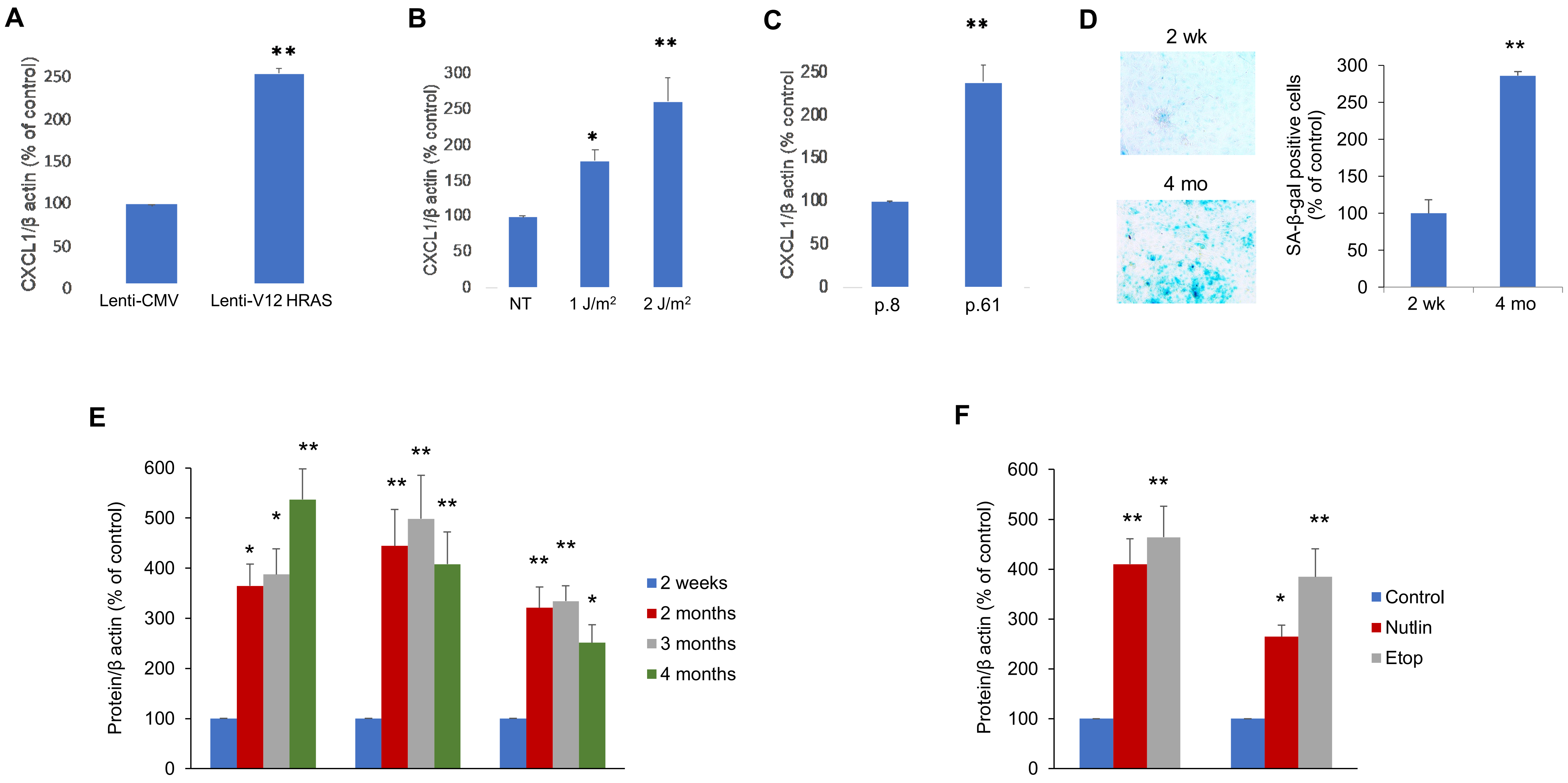
A
CXCL1/β actin (% of control)
0
50
100
150
200
250
**
Lenti-CMV
Lenti-V12 HRAS
B
CXCL1/β actin (% control)
0
50
100
150
200
250
300
*
**
NT
1 J/m2
2 J/m2
C
CXCL 1/β actin (% control
0
50
100
150
200
250
**
p.8
p.61
D
2 wk
4 mo
SA-β-gal positive cells
(% of control)
0
50
100
150
200
250
300
**
2 wk
4 mo
E
Protein/β actin (% of control)
0
100
200
300
400
500
600
*
*
**
**
**
**
**
**
*
CXCL1
β gal
GH
2 weeks
2 months
3 months
4 months
F
Protein/β actin (% of control)
0
100
200
300
400
500
600
**
**
*
**
CXCL1
CXCR2
Control
Nutlin
Etop

Figure S6

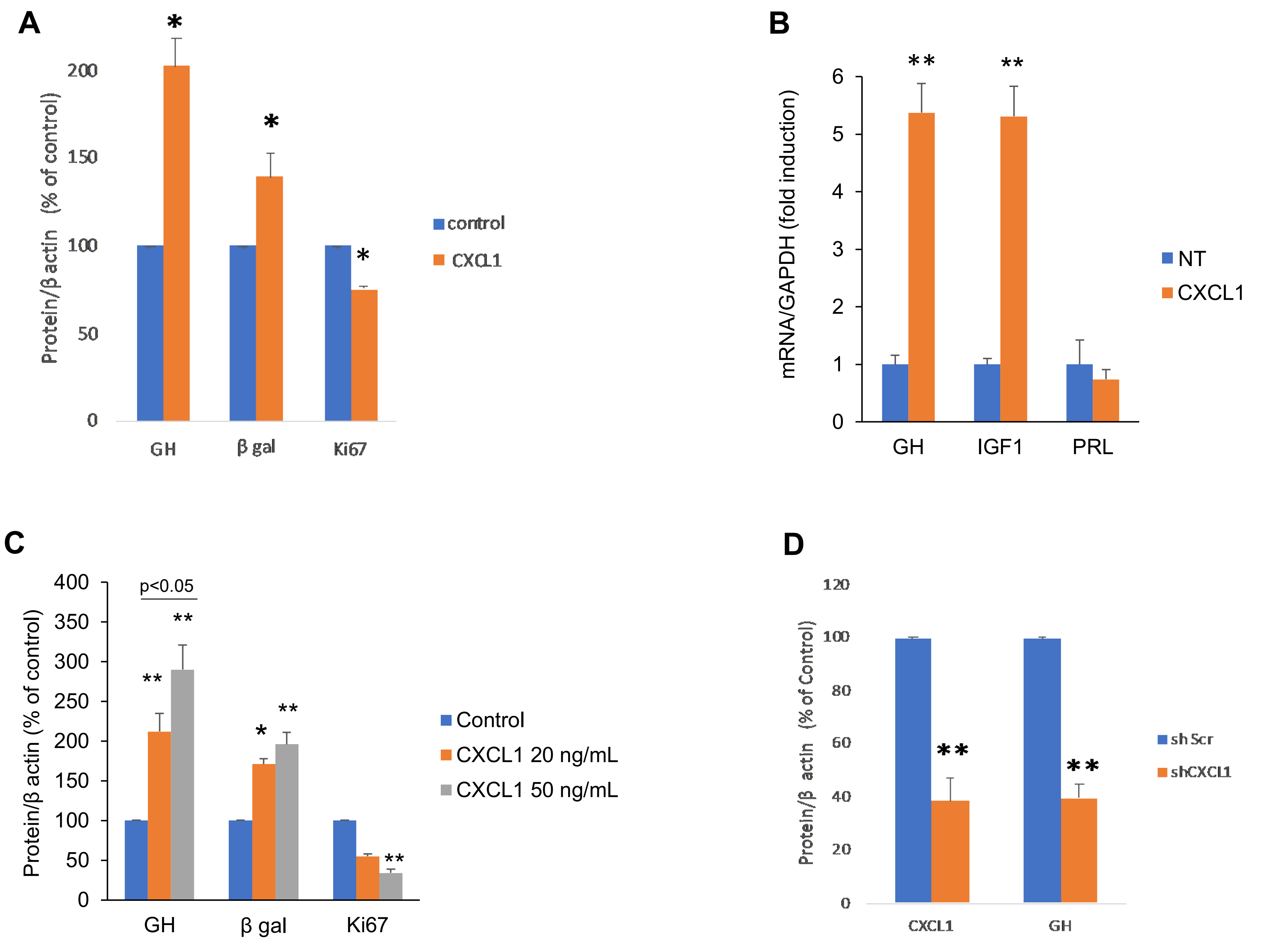
A
Protein/β actin (% of control)
0
50
100
150
200
GH
β gal
Ki67
*
*
*
control
CXCL1
B
mRNA/GAPDH (fold induction)
0
1
2
3
4
5
6
GH
IGF1
PRL
**
**
NT
CXCL1
C
Protein/β actin (% of control)
0
50
100
150
200
250
300
350
400
p<0.05
**
**
*
**
**
GH
β gal
Ki67
Control
CXCL1 20 ng/mL
CXCL1 50 ng/mL
D
Protein/β actin (% of Control)
0
20
40
60
80
100
120
**
**
CXCL1
GH
sh Scr
shCXCL1

Figure S7

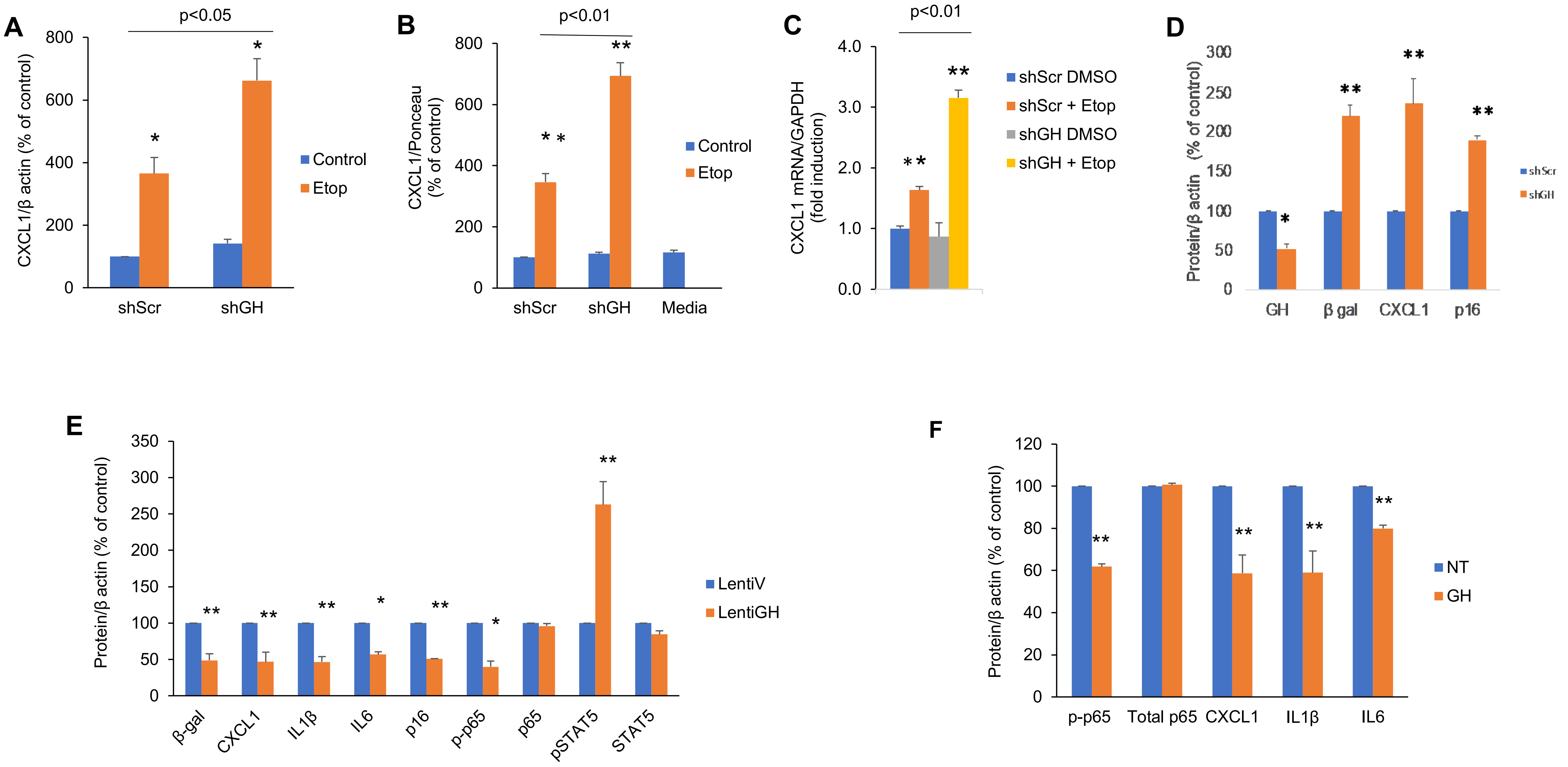
A
p<0.05
CXCL1/β actin (% of control)
800
600
400
200
0
*
*
Control
Etop
shScr
shGH
B
p<0.01
CXCL1/Ponceau (% of control)
800
600
400
200
0
* *
**
Control
Etop
shScr
shGH
Media
C
p<0.01
CXCL1 mRNA/GAPDH (fold induction)
4.0
3.0
2.0
1.0
0.0
**
* *
shScr DMSO
shScr + Etop
shGH DMSO
shGH + Etop
D
Protein/β actin (% of control)
300
250
200
150
100
50
0
*
**
**
**
shScr
shGH
GH
β gal
CXCL1
p16
E
Protein/β actin (% of control)
350
300
250
200
150
100
50
0
**
**
**
*
**
*
**
LentiV
LentiGH
β-gal
CXCL1
IL1β
IL6
p16
p-p65
p65
pSTAT5
STAT5
F
Protein/β actin (% of control)
120
100
80
60
40
20
0
**
**
**
**
NT
GH
p-p65
Total p65
CXCL1
IL1β
IL6

Figure S8

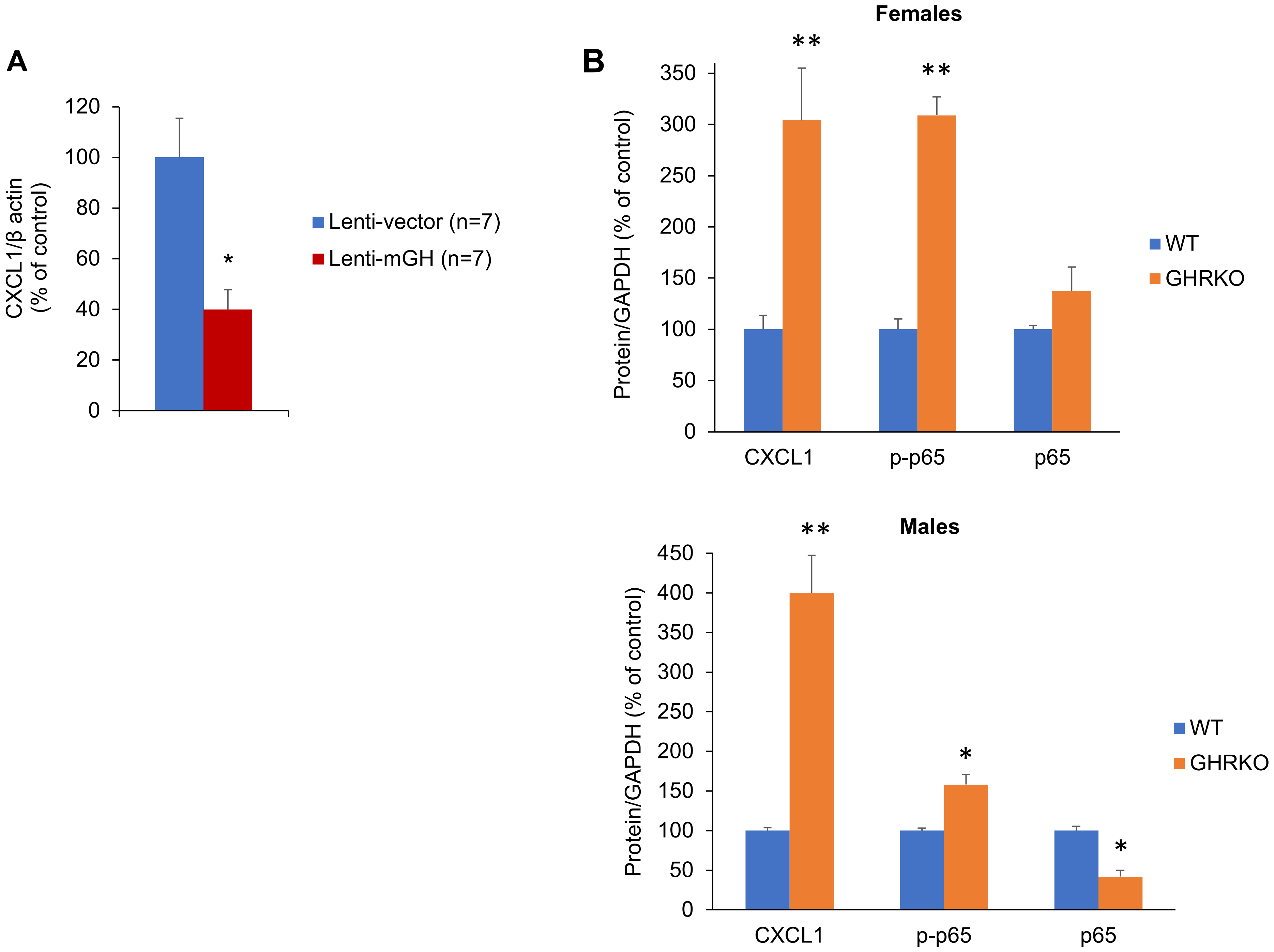
A
CXCL1/β actin (% of control)
0
20
40
60
80
100
120
*
Lenti-vector (n=7)
Lenti-mGH (n=7)
B
Females
Protein/GAPDH (% of control)
0
50
100
150
200
250
300
350
**
**
CXCL1
p-p65
p65
WT
GHRKO
Males
Protein/GAPDH (% of control)
0
50
100
150
200
250
300
350
400
450
**
*
*
CXCL1
p-p65
p65
WT
GHRKO

Figure S9

	Number of tumors observed				Average tumor size (mm)
	**Spleen**	**Liver**	**Peritoneum**	**Total Tumors**	
**HCT116 lenti-Vector (n=8)**	4	0	2	6	1.33 ± 0.18
**HCT116 lenti-hGH (n=8)**	7	4	16	27	2.85 ± 0.43

Table S1

Supplement Figures Legends

Figure S1. GH is induced in different senescence models. (A-C) ImageJ quantification of GH and senescence markers in hNCC. (A) Oncogene-induced senescence. Cells were infected with lentivirus expressing constitutively activated HRAS oncogene (lentiV-12RAS) or empty vector (lenti-CMV) and analyzed 7 days later. (C) Replicative senescence. (B) DNA damage-induced senescence. Cells were exposed to UVC light at indicated doses or left untreated (NT) and analyzed 6 days later. Cells were passaged until proliferation was exhausted. Cells from passage 8 (p.8) and passage 61 (p.61) were compared. **(D-F)** Representative images of SA-β-gal expression in each senescence model. **(G-I)** Number of SA-β-gal-positive cells depicted as percent of control in each senescence model. At least 5 fields were analyzed. **(J)** Telomere length assay showing telomere shortening with prolonged culturing, as a reflection of aging. The results shown as mean ± SEM. In A,C,G,I,J the differences from control were analyzed by two-tail Student's t-test. In B, the differences were analyzed by two-way ANOVA, and in H by one-way ANOVA followed by ad-hoc Tukey's test to adjust for multiple comparisons. In A-C and G-I, results are graphed as percent of control, but statistical testing was performed on raw numbers. *, $p<0.05$; **, $p<0.01$ vs comparator.

Figure S2. GH is induced in senescent cells and is a SASP component. (A) ImageJ quantification of senescence markers in hNCC treated with 50 μM etoposide (Etop) or DMSO for 48h, and analyzed 7 days after beginning treatment. **(B)** Western blot of GH, p16 and PCNA expression and **(C)** Real time PCR results of GH and IGF1 in hNCC stably infected with lenti shScr (as control) or lenti shGH and treated with 50 μM

etoposide (Etop) or DMSO for 48h, and grown for an additional 4 days. In C, results are expressed as fold-change vs control (shScr DMSO treated) taken as 1. Results are shown as mean ± SEM of triplicate measurements. **(D)** ImageJ quantification of secreted GH in culture medium. EM, empty medium. **(E-G)** Senescent markers and IGF1mRNA in fibroblasts derived from patients with progeroid syndrome and healthy volunteers (control). (E) ImageJ quantification of senescence markers. (F) Real-time PCR of IGF1 mRNA. (G) The average of GH and IGF1 mRNA levels from fibroblasts derived from patients with progeroid syndrome and healthy volunteers (control). Real-time PCR results are shown as mean ± SEM of triplicate measurements and expressed as fold-change vs control taken as 1. AT, ataxia telangiectasia; CS, Cockayne syndrome; CSB, Cockayne syndrome group B; HGPS, Hutchinson-Gilford progeroid syndrome; XP, xeroderma pigmentosa. Results are shown as mean ± SEM of triplicate measurements. In B and D, differences were analyzed by two-way ANOVA followed by ad-hoc Tukey's test to adjust for multiple comparisons. In A,E,G differences from control were analyzed by two-tailed Student's t test. In A,B,D,E proteins are normalized to loading control and graphed as percent of control, but statistical testing was performed on raw numbers. *, $p<0.05$; **, $p<0.01$ vs comparator.

Figure S3. GH triggers proliferation in senescent cells. (A) Representative images of non-senescent parental hNCC not treated (NT) and treated with GH only. Ki67 (brown) expression in senescent (blue) SA-β-gal-positive cells. Cells treated with etoposide only or with etoposide and GH are depicted in Figure 2A. **(B)** Senescence markers of hNCC treated with 50 μM etoposide for 48 hours and analyzed 6 days after beginning treatment. **(C)** BrdU incorporation. hNCC that reached replicative

senescence (p.61) were treated with 500 ng/mL GH or left untreated (NT) and analyzed 24h later. **(D)** BrdU incorporation in senescent cells treated with 50 µM etoposide for 48h to induce senescence, then with 500 ng/mL GH for 24h. In C,D results are shown as mean ± SEM of 3 independent experiments and analyzed with two-tail Student test. *, $p<0.05$ control.

Figure S4. GH suppresses p53 in senescent cells and affects colony formation. (A-B) ImageJ quantification of p53 in (A) hNCC infected with either lenti-shScr as control or lenti-shGH, treated with 50 µM etoposide (Etop) or left untreated (Control) for 48h, and grown for an additional 4 days; and (B) hNCC pre-treated with 500 ng/mL GH and indicated doses of etoposide, then analyzed 24h later. Results are shown as mean ± SEM of at least 3 replicate measurements normalized to loading control. Differences were analyzed in A by one-way ANOVA and in B by two-way ANOVA followed by ad-hoc Tukey's test to adjust for multiple comparisons. **(C)** Western blot of GH and 53 in colon tissue of hypophysectomized rats after 5 injection of either Oxaliplatin (Oxa) (i.p. 4 mg/kg) alone or oxaliplatin with pegvisomant (PEG) (s.q. 0.57 mg/kg). Lower panel: ImageJ quantification of GH and p53. For (A-C) C, control. **(D-F)** hNCC were pretreated with 50 µM etoposide for 48h, sorted for senescence, and cultured in the presence of 500 ng/mL GH 10 days after plating. (D) Number of colonies per well. (E) Western blot of cleaved caspase 3. (F) Colony size. One dot represents one experiment. In C and E, results are shown as mean ± SEM. In C, D and F results were analyzed by two tailed Student's t test. Results are graphed as percent of control, but statistical testing was performed on raw numbers. *, $p<0.05$; **, $p<0.01$ vs control.

Figure S5. Paracrine GH triggers proliferation and EMT and suppresses DNA damage pathway. (A) ImageJ quantification of proliferation, stemness, and EMT markers in hNCC line #1 treated with 50 μM etoposide, sorted for senescence 7 days later, and cultured for 10 days in the presence of or absence of GH; cells were then sorted again for SA-β-gal positivity. Only SA-β-gal negative (post-senescent cells) were analyzed. **(B)** Organoids were infected with lentiV or lentiGH (both expressing GFP) and cultured for 5 weeks; organoid cells were then sorted for GFP-positive and GFP-negative expression and only GFP-negative cells co-cultured with either lentiV or lentiGH organoid cells were analyzed. ImageJ quantification of DNA damage and repair proteins shown. In A and C, results are shown as mean ± SEM of triplicate measurements normalized to loading control. In A, results were analyzed by one-way ANOVA followed by ad-hoc Tukey's test to adjust for multiple comparisons. In C, results were analyzed by two-tailed Student's t-test. Results are graphed as percent of control, but statistical testing was performed on raw numbers. *, $p<0.05$; **, $p<0.01$ vs comparator.

Figure S6. CXCL1 is induced in senescent cells. (A-C) Image J quantification of CXCL1 in different models of senescence. (A) Oncogene-induced senescence. Cells were infected with lentivirus expressing constitutively activated HRAS oncogene (lentiV12HRAS) or empty vector (lentiV-CMV) and analyzed 7 days later. (B) DNA damage induced senescence. Cells were exposed to indicated doses of UVC light or left untreated (NT) and analyzed 6 days later. (C) Replicative senescence. Cells were passaged until proliferation was significantly reduced. Cells from passage 8 (p.8) and passage 61 (p.61) were compared. In all experiments, CXCL1 expression was normalized

to the same loading control as in Figures 1A, 1B, and 1C, respectively. (D) SA-β-gal enzymatic activity in human intestinal organoids cultured for 2 weeks and 4 months. Representative images and number of SA-β-gal–positive cells depicted as percent of control. Six fields/experiment were counted. Results are shown as mean ± SEM. **(E)** ImageJ quantification of senescent organoid proteins cultured for up to 4 months. **(F)** ImageJ quantification of CXCL1 and its receptor CXCR2 in hNCC 72h after treatment with either 3 μM nutlin3 (Nutlin) or 5 μM etoposide (Etop) for 48h or left untreated (Control, C). In A-F, results are shown as mean ± SEM normalized to loading control. In A,C,D differences between cultures was analyzed with two-tailed Student's t-test. In B,E,F differences were analyzed by one-way ANOVA followed by ad-hoc Tukey's test to adjust for multiple comparisons. Results are graphed as percent of control, but statistical testing was performed on raw numbers. *, $p<0.05$; **, $p<0.01$ vs comparator.

Figure S7. CXCL1 induces GH expression. (A) ImageJ quantification of proteins from organoids treated with 100 ng/mL CXCL1 for 96h. **(B)** Real-time PCR of senescent organoids treated with CXCL1 for 96h. Experiment was repeated 3 times. In A and B, differences with control (NT) were analyzed with two-tailed Student's t-test. In B, results are shown as mean ± SEM of triplicate measurements, expressed as fold-change vs control taken as 1. **(C)** ImageJ quantification of hNCC treated with indicated doses of CXCL1 for 96h. **(D)** ImageJ quantification of hNCC infected with lenti-shCXCL1 or lenti-shScr and analyzed 3 days after infection. In C and D, results were analyzed by one- and two-way ANOVA, respectively, followed with ad-hoc Tukey's test to adjust for multiple comparisons. Results are shown as mean ± SEM of at least 3 replicate measurements normalized to loading controls. Results are graphed as percent of

control, but statistical testing was performed on raw numbers. *, $p<0.05$; **, $p<0.01$ vs comparator.

Figure S8. GH suppresses CXCL1 expression and secretion in senescent cells. (A-C) hNCC were infected with either lenti-shGH or lenti-shScr, treated with 50 μM etoposide (Etop) to induce senescence for 48h or left untreated (C) and analyzed 6 days later. (A) ImageJ quantification of CXCL1 in cells. (B) ImageJ quantification of CXCL1 in culture medium. (C) Real-time PCR of CXCL1 mRNA. In A-C, differences with respective controls were analyzed by two-way ANOVA followed by ad-hoc Tukey's test to adjust for multiple comparisons. Results are shown as mean ± SEM of triplicate measurements and in C are depicted as fold-change vs control taken as 1. **(D)** ImageJ quantification of proteins in organoids 1 week after infection with lenti-shGH or lenti-shScr. **(E)** ImageJ quantification of proteins in organoids 1 week after infection with lentiGH or lentiV. **(F)** Image J quantification of proteins in hNCC treated with 50 μM etoposide for 2 days, sorted for senescence on day 3, and treated with GH for an additional 24h. Results are shown as mean ± SEM of triplicate measurements normalized to loading control. In D-F, results were analyzed by two-tailed Student's t-test. Results are graphed as percent of control, but statistical testing was performed on raw numbers. *, $p<0.05$; **, $p<0.01$ vs comparator.

Figure S9. GH suppresses CXCL1 and GH deficiency upregulates CXCL1 in vivo. (A) GH suppresses CXCL1 in vivo. ImageJ quantification of CXCL1 in colon tissue from female nude mice implanted with HCT116 GH-secreting xenografts (lenti-mGH) or empty vector (lenti-V) and sacrificed 5 weeks later. **(B)** GH signaling deficiency results

in CXCL1 upregulation in vivo. ImageJ quantification of CXCL1, p-p65, and p65 in colon tissue of female and male GHR knockout (GHRKO) and wild-type (WT) mice. Results are shown as mean ± SEM normalized to loading control, assessed using two-tailed Student's t test. *, $p<0.05$; **, $p<0.01$ vs control.

Table S1. Metastatic lesions developed in Nu/J mice carrying GH-secreting (lentiGH) or control vector-expressing (lentiV) xenografts 4 weeks after intrasplenic injection of post-senescent hNCC.
